# Discovery of Zilucoplan: A Complement C5 Inhibitor for Treatment of Anti-Acetylcholine Receptor (AChR) Antibody-Positive Generalized Myasthenia Gravis (gMG)

**DOI:** 10.1021/acs.jmedchem.5c02537

**Published:** 2025-12-11

**Authors:** Ping Ye, Robert P. Hammer, Zhaolin Wang, Ketki Dhamnaskar, Michelle Hoarty, Zhong Ma, Guo-Qing Tang, Steven J. DeMarco, Alonso Ricardo

**Affiliations:** † UCB Bioscience, Cambridge, Massachusetts 02140, United States; ‡ Ra Pharmaceuticals, Cambridge, Massachusetts 02140, United States

## Abstract

Complement component 5 (C5) is a protein in the complement cascade and a part of the innate immune system that has been clinically validated as a therapeutic target for several immune-mediated diseases including generalized myasthenia gravis (gMG). In this paper, we discuss the early discovery of zilucoplan, a macrocyclic peptide drug, which was identified via innovative extreme diversity mRNA display ( MaZ., ; HartmanM. C. T., In Vitro Selection of Unnatural Cyclic Peptide Libraries via mRNA Display. In Ribosome Display and Related Technologies: Methods and Protocols; DouthwaiteJ. A., , JacksonR. H., , Eds.; Springer: New York, 2012; pp 367−390.10.1007/978-1-61779-379-0_2122094817) against C5 and approved for the treatment of gMG. We highlight the key steps and rationale behind the peptide medicinal chemistry optimization of the initial screening hits, that led to improved potency, stability, and pharmacokinetic properties.

## Graphical abstract

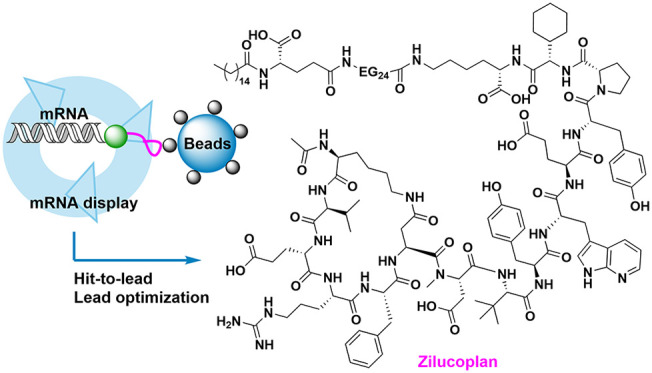

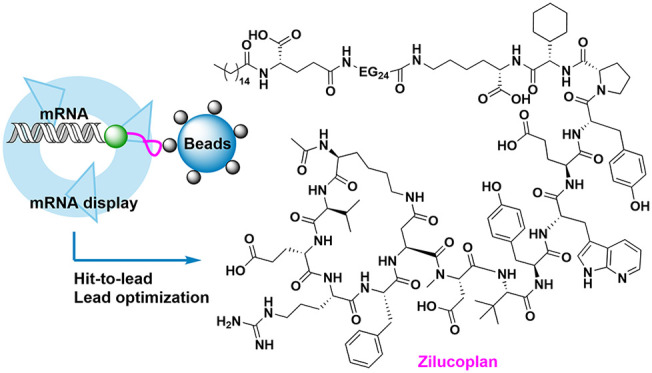

## Introduction

The complement system plays a crucial role in the innate immune response, and its dysregulation contributes to various autoimmune diseases, such as paroxysmal nocturnal hemoglobinuria (PNH), atypical hemolytic uremic syndrome (aHUS), and generalized myasthenia gravis (gMG).^1–4^ There are three complement pathways: the classical pathway, the alternative pathway and the lectin pathway, each with their own unique activation process.^5^ The classical pathway is triggered when C1q binds to an antibody/antigen complex. The alternative pathway is constitutively activated at a low level via C3 hydrolysis. The lectin pathway is activated by binding of mannose-binding lectin to a pathogen surface. Activation of these pathways results in cleavage of C5 to C5a and C5b. C5a is an important chemotactic protein that helps recruit inflammatory cells. C5b initiates the formation of the membrane attack complex (MAC), consisting of C5b, C6, C7, C8, and multiple C9. MAC is the cytolytic end-product of the complement cascade. It forms a transmembrane channel, which causes osmotic lysis of the target cell.^6,7^

gMG is an autoimmune disease in which pathogenic autoantibodies damage the neuromuscular junction, resulting disabling or potentially life-threatening muscle weakness.^8^ About 80% of gMG patients are antiacetylcholine receptor (AChR) antibody-positive.^9^ The antibodies can block AChR at the neuromuscular junction, preventing nerve impulses from triggering muscle contractions. In addition, most of these antibodies, are immunoglobulin G (IgG) IgG1 and IgG3 subclasses, which can activate complement and form the membrane attack complex (MAC), leading to postsynaptic membrane damage and muscle weakness.^10^

Several C5 inhibitors have been approved or are under development for the treatment of autoimmune diseases, including the monoclonal antibody eculizumab^3,4,11,12^ and other antibodies,^13^ an RNA aptamer^14^ and small molecule inhibitors.^15^

Herein we describe the discovery of zilucoplan, a synthetic macrocyclic peptide that specifically binds to human complement C5 with high affinity, blocking its cleavage by C5 convertases and the assembly of the cytolytic membrane attack complex. Zilucoplan effectively prevents the activation of C5 clinical wild-type and polymorphic R885 variants. Patients with these R885 variants have been reported to respond poorly to some of the approved antibody-based treatment.^16^ Zilucoplan also interferes with the formation of C5b6 and inhibits red blood cell (RBC) hemolysis induced by plasmin-mediated noncanonical C5 activation.^17^ Zilucoplan has been approved in multiple countries for the treatment of adults with anti-AChR antoantibody-positive gMG and is given as a self-administered daily subcutaneous injection.^18–20^

## Results and Discussion

### Screening and Structure–Activity Relationship

We used mRNA display technology^1,21^ as a tool to identify peptide inhibitors of C5 that could be optimized using traditional medicinal chemistry into a drug candidate. The use of mRNA display has proved to be a powerful platform for hit finding,^1,21,22^ as previously exemplified by the discovery of an orally available proprotein convertase subtilisin/kexin type 9 (PCSK9) inhibitor which recently announced positive topline results from a Phase 3 clinical trial.^23,24^ Below, we describe our approach for hit optimization focused on initial potency improvements, stabilization of metabolic soft spots, and finally engineering of in vivo half-life to support the development of zilucoplan as a daily, subcutaneous self-administered drug.^18–20^

Screening of two different mRNA libraries generated distinct hit series (Figure 1). Compound **1** was a potent 13-amino acid linear peptide, while Compound **2** was less potent but contained two cysteines which allowed for direct cyclization using a xylene bromide cross-linking reagent. Comparison of the two series showed high homology, which inspired the design of a hybrid of the two series and led to increased plasma stability. The C-terminal lipidation improved PK properties through albumin binding.^25^ The rationale of including an ethylene glycol spacer was to minimize potential interference from albumin binding on target engagement. This paper will focus on the optimization of the initial mRNA hit compound 1, using standard empirical chemistry strategies, which led to the later approved drug.

**Figure 1:**
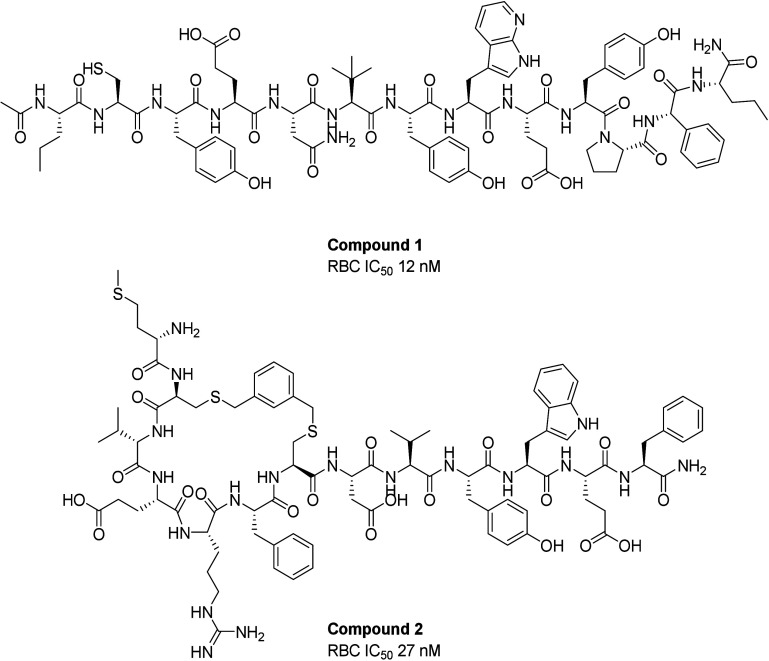
Structures of compounds **1** and **2**.

The peptides were evaluated for C5 inhibition activity using a red blood cell (RBC) lysis assay (described in the Experimental Section), and the results were reported as half-maximum inhibitory concentration (IC_50_) values. In general, the peptides were synthesized using standard 9-fluorenylmethoxycarbonyl (Fmoc) solid-phase linear peptide synthesis (SPPS), followed by in-solution or on-resin cyclization. Specific synthetic details are provided in the Experimental Section.

Compound **1** had some chemical liabilities. The cysteine (Cys) residue at position 2 was susceptible to oxidation, resulting in dimer formation. Replacement of Cys with serine (Ser) (compound **3**, Table 1) and Norvaline (Nvl) (compound **15**, Table 2) were well tolerated, with little impact on complement inhibition (IC_50_ of 10 nM and 14 nM, respectively). The phenylglycine (Phg) at position 12 is notorious for epimerization under standard peptide coupling and Fmoc deprotection conditions, resulting in close-eluting diastereomers, which are challenging for characterization and separation. Replacement of Phg with cyclohexylglycine (Chg) was tolerated resulting in an IC_50_ of 14 nM (compound **4**, Table 1).

**Table 1:** Alanine Scan and Modifications to Compound **1** ^a^

Compound	IC_50_ (nM)^b^	P1	P2	P3	P4	P5	P6	P7	P8	P9	P10	P11	P12	P13
**1**	12	Nvl	C	Y	E	N	Tle	Y	AzaTrp	E	Y	P	Phg	Nvl
**3**	10	Nvl	**S**	Y	E	N	Tle	Y	AzaTrp	E	Y	P	Phg	Nvl
**4**	14	Nvl	C	Y	E	N	Tle	Y	AzaTrp	E	Y	P	**Chg**	Nvl
**5**	>10,000	Nvl	S	**A**	E	N	Tle	Y	AzaTrp	E	Y	P	Chg	Nvl
**6**	161	Nvl	S	Y	**A**	N	Tle	Y	AzaTrp	E	Y	P	Chg	Nvl
**7**	54	Nvl	S	Y	E	**A**	Tle	Y	AzaTrp	E	Y	P	Chg	Nvl
**8**	90	Nvl	S	Y	E	N	**A**	Y	AzaTrp	E	Y	P	Chg	Nvl
**9**	104	Nvl	S	Y	E	N	Tle	**A**	AzaTrp	E	Y	P	Chg	Nvl
**10**	>10,000	Nvl	S	Y	E	N	Tle	Y	**A**	E	Y	P	Chg	Nvl
**11**	>10,000	Nvl	S	Y	E	N	Tle	Y	AzaTrp	**A**	Y	P	Chg	Nvl
**12**	>10,000	Nvl	S	Y	E	N	Tle	Y	AzaTrp	E	**A**	P	Chg	Nvl
**13**	123	Nvl	S	Y	E	N	Tle	Y	AzaTrp	E	Y	**A**	Chg	Nvl
**14**	128	Nvl	S	Y	E	N	Tle	Y	AzaTrp	E	Y	P	**A**	Nvl

a All peptides were C-terminal amides and N-terminal acetylated.

b C5 inhibition activity using a red blood cell (RBC) lysis assay described in the Experimental Section, and the results were reported as half-maximum inhibitory concentration (IC_50_) values. All values were the average of triplicates.

**Table 2:** *N*-Methylation Scan on Compound **15** ^a^

Compound	IC_50_ (nM)^b^	P1	P2	P3	P4	P5	P6	P7	P8	P9	P10	P11	P12	P13
**15**	14	Nvl	Nvl	Y	E	N	Tle	Y	AzaTrp	E	Y	P	Phg	Nvl
**16**	37	Nvl	Nvl	**N-MeY**	E	N	Tle	Y	AzaTrp	E	Y	P	Chg	Nvl
**17**	2780	Nvl	Nvl	Y	**N-MeE**	N	Tle	Y	AzaTrp	E	Y	P	Chg	Nvl
**18**	12	Nvl	Nvl	Y	E	**N-MeN**	Tle	Y	AzaTrp	E	Y	P	Chg	Nvl
**19**	>10,000	Nvl	Nvl	Y	E	N	Tle	**N-MeY**	AzaTrp	E	Y	P	Chg	Nvl
**20**	441	Nvl	Nvl	Y	E	N	Tle	Y	**N-MeW** ^**b**^	E	Y	P	Chg	Nvl
**21**	>10,000	-	-	Y	E	N	Tle	Y	AzaTrp	**N-MeE**	Y	P	Phg	Nvl
**22**	>10,000	Nvl	Nvl	Y	E	N	Tle	Y	AzaTrp	E	**N-MeY**	P	Chg	Nvl
**23**	804	Nvl	Nvl	Y	E	N	Tle	Y	AzaTrp	E	Y	P	**N-MePhg**	Nvl

a All peptides were C-terminal amides and N-terminal acetylated; b. N-Me Trp as the surrogate of N-Me AzaTrp

b C5 inhibition activity using a red blood cell (RBC) lysis assay described in the Experimental Section, and the results were reported as half-maximum inhibitory concentration (IC_50_) values. All values were the average of triplicates.

Alanine (Ala) scanning was performed using a version of the peptide with Ser and Chg replacements on the scaffold. Results are summarized in Table 1. The Ala scan suggested that the aromatic residues at positions 3, 8, and 10 and the Glu at position 9 were key residues for interacting with C5, Ala substitutions at any of those positions resulted in complete loss of potency (compounds **5**, **10**, **11**, and **12**). Ala substitutions at other positions in the linear peptide also reduced binding but were somehow tolerated with less than a 15-fold loss of potency.

In parallel efforts, a backbone *N*-methylation scan was performed using Cys to norvaline (Nvl) replacement (compound **15**) as the scaffold. Results are summarized in Table 2. *N*-Methylation was not tolerated, except for the amino acid residues at positions 3 (compound **16**, IC_50_ = 37 nM) and 5 (compound **18**, IC_50_ = 12 nM). This modification will later become important during stability optimization of the lead series.

In an attempt to reduce the size and molecular weight of the target peptides, truncations from the N and C termini were performed using compound **1** as the scaffold. Results are summarized in Table 3. Truncation of N- or C-terminal Nvl had little or no impact on the potency (compound **24**: IC_50_ 32 nM; compound **28**: IC_50_ 18 nM). The C-terminal Nvl corresponds to the last variable residue of the mRNA screening library (before fixed linker-mRNA) and was identified as an ideal site for the replacement or conjugation of residues that could modulate solubility or pharmacokinetic properties. Truncation further beyond Nvl from either terminus showed a progressive loss of potency. Small linear peptides with truncations of more than three residues, compared to the parent molecule, did not show measurable C5 inhibition activity (compounds **30** and **31**).

**Table 3:** Truncation Scan on Compound **1** ^a^

Compound	IC_50_ (nM)^b^	P1	P2	P3	P4	P5	P6	P7	P8	P9	P10	P11	P12	P13
**1**	12	Nvl	C	Y	E	N	Tle	Y	AzaTrp	E	Y	P	Phg	Nvl
**24**	32	-	C	Y	E	N	Tle	Y	AzaTrp	E	Y	P	Phg	Nvl
**25**	48	-	-	Y	E	N	Tle	Y	AzaTrp	E	Y	P	Phg	Nvl
**26**	1020	-	-	-	E	N	Tle	Y	AzaTrp	E	Y	P	Phg	Nvl
**27**	>10,000	-	-	-	-	N	Tle	Y	AzaTrp	E	Y	P	Phg	Nvl
**28**	18	Nvl	C	Y	E	N	Tle	Y	AzaTrp	E	Y	P	Phg	-
**29**	84	Nvl	C	Y	E	N	Tle	Y	AzaTrp	E	Y	P	-	-
**30**	>10,000	-	-	-	-	-	Tle	Y	AzaTrp	E	Y	-	-	-
**31**	>10,000	-	-	-	-	-	-	Y	AzaTrp	E	Y	P	-	-

a All peptides were C-terminal amides and N-terminal acetylated.

b C5 inhibition activity using a red blood cell (RBC) lysis assay described in the Experimental Section, and the results were reported as half-maximum inhibitory concentration (IC_50_) values. All values were the average of triplicates.

### Discovery of a Hybrid Peptide

A plasma stability assay showed poor stability of compound **1** (4% remaining after 24 h in mouse plasma; 9% remaining after 24 h in human plasma) and attempted cyclization of linear compound **1** to enhance its stability, were not successful (data not shown). Inspired by the structural similarity between compounds **1** and **2**, a novel hybrid strategy was explored. The hybrid compound consisted of the N-terminal cyclic “head” of compound **2** (green color, Figure 2), and the C-terminal “tail” of compound **1** (peach color, Figure 2). The hybrid molecule contains 15 AA, with key aromatic residues renumbered as P5, P10, and P12 and polar residue P11, with P5 within the ring. As expected, compound **32** maintained good potency (IC_50_ = 9 nM) and showed good plasma stability in human plasma (95% remaining after 24 h). The poor rat plasma stability (2% remaining after 24 h) was believed to be due to species-specific enzymatic degradation. The analogues of Phg replacement with Chg were equally potent as their parent compound (compound **33**, IC_50_ = 11 nM, Figure 3). Compound **32** became a key analogue of our optimization program.

**Figure 2:**
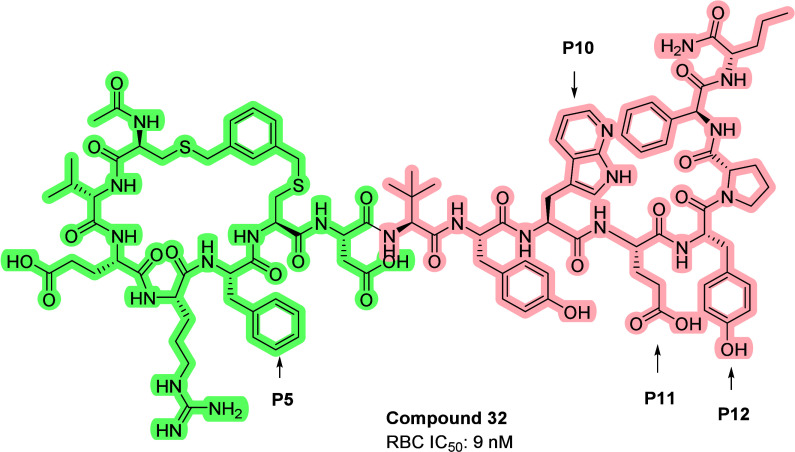
Structure of compound **32**.

**Figure 3:**
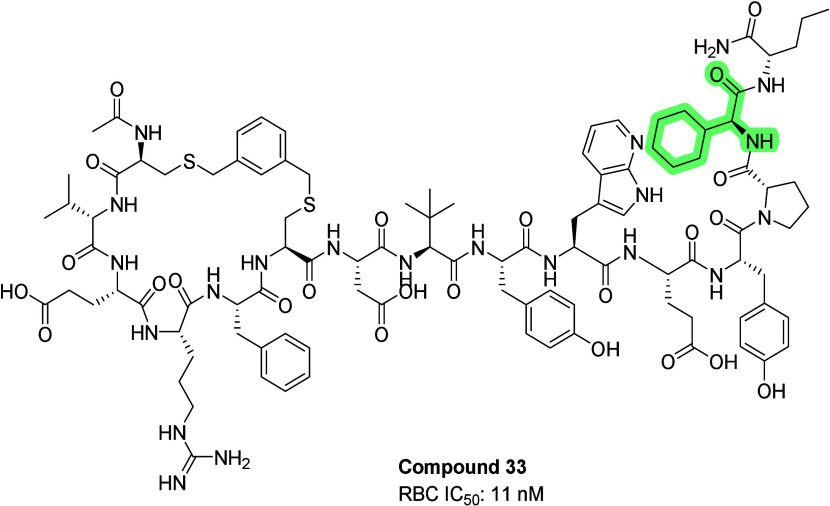
Structure of compound **33**.

### Discovery of an Amide Linker to Replace the Xylene Thioether Linker

The exploration of xylene thioether linker replacement for Compound **32**, yielded several interesting results, as illustrated in Figure 4. Replacing xylene with disulfide resulted in approximately a 2-fold loss of potency (compound **34**, IC_50_ = 17 nM), but demonstrated that the ring size was not critical factor for activity against the target. N-acetyl truncation of the xylene linker had almost no impact on potency (compound **35**, IC_50_ = 8 nM). A thioether linker increased the potency about 2.7-fold (compound **36**, IC_50_ = 4 nM), while a lactam linker through Lys and Asp also increased the potency about 2.2-fold (compound **37**, IC_50_ = 5 nM). To avoid redox liabilities with sulfur containing analogs, we decided to adopt the lactam linker as the cyclization moiety.

**Figure 4:**
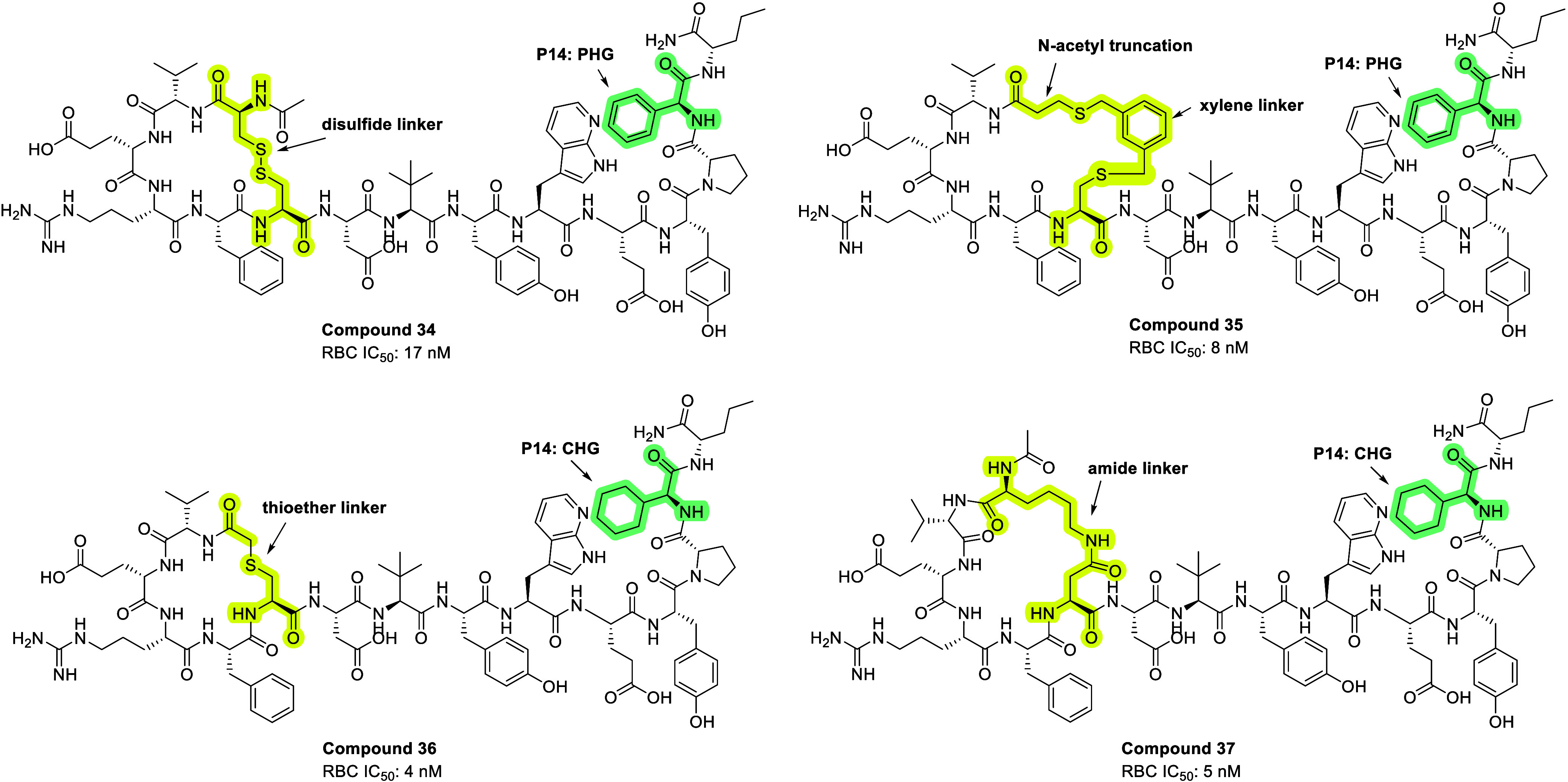
Structures of xylene linker replacement analogues, linkers are highlighted yellow. The rest of the molecules are identical except for position 14, highlighted in green.

### Metabolic Identification Studies

The metabolic identification study of compound **37** identified a proteolytic cleavage site just outside the ring, the amide bond on the N-terminus of position 7 Asp. Deamination of the C-terminal amide was also observed (which was very prominent in rat serum; data not shown). To address these issues, modified analogues were synthesized with alpha-methyl Asp, tetrazole analog and *N*-methyl Asp at position 7 to block the proteolytic cleavage. All these analogs retained inhibition activity and *N*-methyl Asp mapped to the former *N*-methylated analog of peptide **15** at position 5, compound **18**. An analog with an *N*-methyl Asp and C-terminal acid was synthesized to mitigate the aforementioned metabolic degradations (compound **38**, Figure 5). The primary RBC inhibition assay showed increased potency (IC_50_ = 0.9 nM). Compound **38** was selected as a candidate for an in-depth characterization. It showed tight binding to full length human C5 and C5-TED domain by surface plasmon resonance (SPR), with binding affinity (*K*_D_) values of 0.4 and 0.7 nM, respectively. Plasma stability showed 84%, 62%, and 88% remaining after 4 h in human, monkey, and rat plasma, respectively. PK studies were conducted by Charles River Laboratories and analyzed by Agilux. The compound showed reasonable PK in monkey intravenously (IV; 3 mpk), with half-life of 20.7 h. This data supported further development of the molecule toward the goal of a once-daily subcutaneous dosing regimen.

**Figure 5:**
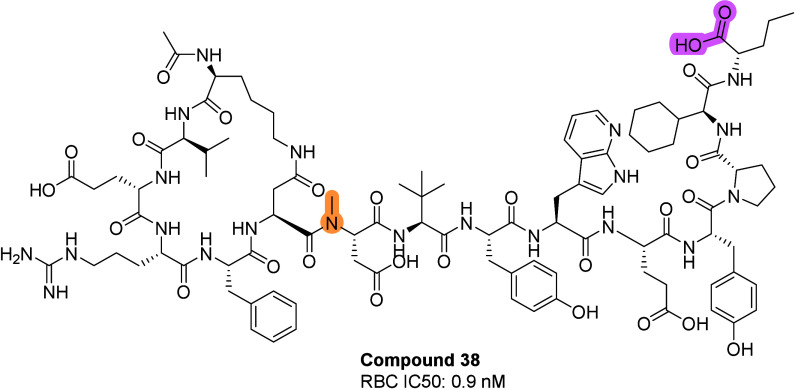
Structure of compound **38**.

### Cocrystallization of Peptide-C 5d Complex

Although a cocrystal structure was not available during structure–activity relationship (SAR) studies to guide the design, we were able to identify compound **38** bound C5 and C5d fragments with equal affinity. With this information, we succeeded at obtaining a cocrystal structure of compound **46** (see structure in ) with the C5d/thioester-containing domain (TED) (Figure 6). Unlike eculizumab, which binds to the MG7 subdomain consisting of R885, the zilucoplan analogue binds to the distant C5d/TED domain and retains the full capacity to bind C5 R885C/H variants and to block their activation.^17^ Modeling the C5d/TED-compound **46** cocrystal structure into the C5b6 structure aligns the cyclic peptide well with the linker between C6 TSP3 and CCP1 subdomains deeply buried in C5b, suggesting steric blockade of C6 by zilucoplan to counteract the formation of C5b6. To be clear, zilucoplan inhibited the binding of C5 to C3b and blocked C5 cleavage to C5a and C5b. This structure explained why the peptide blocked the formation of C5b6 complex and inhibited RBC hemolysis induced by plasmin-mediated noncanonical C5 activation.^17^

**Figure 6:**
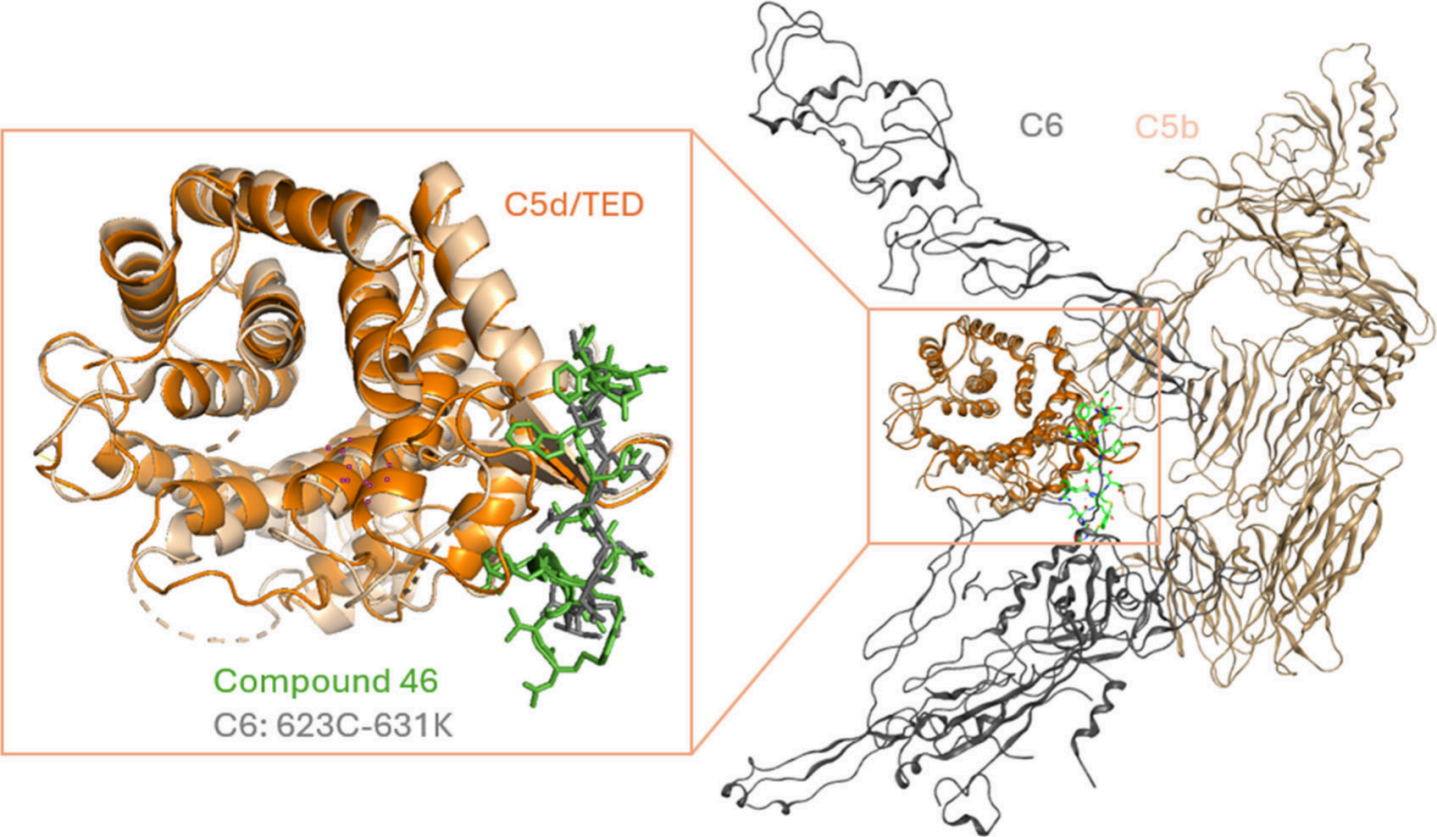
A crystal structure of compound **46** (green), an analogue of zilucoplan, with C5d/TED (orange), superimposed with C5b6 (PDB 4A5W, in which C5b is in golden brown and C6 in gray). Compound **46** in the complex with C5d/TED aligns with C623–K631 of C6, which is part of the linker between TSP3 and CCP1 subdomains and deeply inserted into C5b (schematic structures were generated by MOE2024.06).

### Lipidation to Improve Pharmacokinetic Properties

To enable once-daily dosing and achieve complete and continuous complement control for all patients with anti-AChR antibody-positive gMG, a lipidation strategy was applied to further improve PK through albumin binding.^26^ From a previous Ala scan, it was known that C-terminal Nvl was not required for affinity. Therefore, position 15 was chosen as the site for derivatization, and Lys was chosen as the vector at this position (compound **39**, IC50 2.5 nM, Table 4). A small set of spacer, linker, and protractor combinations were synthesized and tested; results are summarized in Table 4. Without protractors, compounds maintained good potency (compounds **40** and **41**), but no improvement in PK properties (compounds **40**). Compound **42** without a linker showed good potency (IC_50_ = 3.1 nM), but PK properties were not determined. Compound **43**, without the PEG_24_ spacer and gamma glutamic acid (γE) linker, showed approximately a 10-fold loss of potency (IC_50_ = 13 nM), possibly due to interference with target engagement from the steric hindrance effect imposed by albumin binding. Compound **44** utilized a modification from semaglutide but showed about a 40-fold decrease in potency (IC_50_ = 41 nM), possibly due to the combined effect of a short spacer and C18 diacid protractor. Compound **45**, with combined PEG_24_, γE and C16, showed excellent PK while maintaining good potency (IC_50_ = 2.9 nM). It showed tight binding to full length human C5 by SPR, with binding affinity (*K*_D_) values of 0.43 nM. Compound **45** was a potent inhibitor of primate complement and a poor inhibitor of most other species except pigs.^17^ Cynomolgus monkeys were selected for the PK/PD studies to evaluate the inhibitory activity of compound **45** in an animal model. Single dose studies were conducted in cynomolgus monkeys. Compound **45** was administered to male monkeys (*n* = 2 per dosing group) via a single IV or a single SC injection at 0.4 and 0.5 mg/kg. Multidose studies were also conducted in cynomolgus monkeys. Compound **45** was administered to male cynomolgus monkeys (*n* = 2 per dosing group) via daily SC injection at 0.2 and 4 mg/kg. Plasma drug concentrations were determined by LCMS, and the complement activity was assayed using the RBC lysis assay. Results indicated that plasma drug levels should be at or greater than 2.5 μg/mL in monkeys to achieve complete inhibition of hemolysis. At a dose of 4 mg/kg/day, hemolysis was completely inhibited throughout dosing and remained below 3% at 48 h after the last dose (day 9; 216 h). Four days after the final dose, hemolysis reached approximately 10% of the baseline. This result further demonstrated a strong correlation between the pharmacokinetics (PK) and pharmacodynamics (PD) profiles of compound **45** (). This compound became the drug substance of zilucoplan.

**Table 4:**
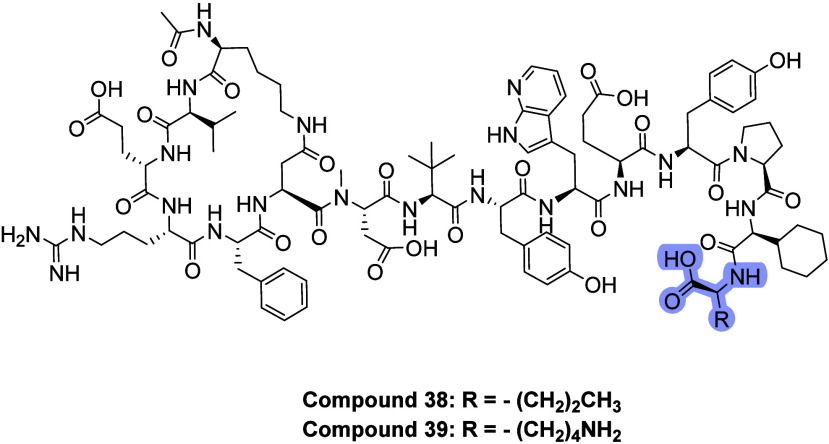
Modifications to Understand PK Properties

	R					Monkey iv PK	
Compound	RBC IC_50_ (nM)^f^	Vector	Spacer	Linker	Protractor	AUC_last_ (h*ng/mL)	*t*_1/2_ (h)
**38**	0.9 ± 0.15 (*n* = 6)	Nvl	-	–	-	20596^c^	20.7
**39**	2.5 ± 0.41 (*n* = 6)	εK	-	–	-	-ND^a^	-ND
**40**	1.4	εK	PEG_24_	γE	-	33205^b^	24
**41**	3.3	εK	PEG_24_	-	-	ND	ND
**42**	3.1	εK	PEG_24_	-	C16	ND	ND
**43**	13	εK	-	-	C16	947192^c^	13^a^
**44**	41	εK	PEG_2‑_PEG_2_	γE	C18OH	562735^d^	-
**45**	2.9 ± 0.8 (*n* = 6)	εK	PEG_24_	γE	C16	429638^e^	182

a ND = not done.

b 1 mg/kg.

c 3 mg/kg.

d 0.5 mg/kg.

e 0.4 mg/kg.

f C5 inhibition activity using a red blood cell (RBC) lysis assay described in the Experimental Section, and the results were reported as half-maximum inhibitory concentration (IC_50_) values. All values were the average of *n* ≥ 3, standard deviation was calculated for key compounds.

g Multiphasic clearance profile. 13 represents the t_1/2_ for earlier phase. The terminal t_1/2_ could not be accurately determined, but is >24 h.

## Conclusions

In summary, we have taken two overlapping hit peptides from mRNA display screens, combined them, and then optimized the hybrid peptide’s potency,plasma stability and pharmacokinetic properties. The molecule has demonstrated complete inhibition of complement activity with adequate safety margins and has been approved by multiple countries for the treatment of adult patients with anti-AChR antibody-positive gMG. It is the first once-daily subcutaneous self-injection therapy for adults with gMG.^18^ It is also the first approved peptide therapeutic derived from an mRNA display screen.^27^

## Experimental Section

### Materials

Amino acid building blocks, solvents and reagents were obtained from various commercial sources such as Sigma-Aldrich, Novabiochem, Chem-Impex, Iris Biotech, and Nagase & Co. Ltd. The noncanonical amino acid Fmoc-AzaTrp and Fmoc-NMeAsp were supplied by Peptech. Boc-γE­(NHPEG_24_CO_2_Pfp)-O*t*Bu was provided by Peptech. The Fmoc-amino-PEG_24_-NHS ester was purchased from Quanta BioDesign and BroadPharm. Palmitic acid NHS ester was purchased from Sigma-Aldrich. (*t*Bu)­C18-γE­(NHPEG_24_CO_2_Pfp)-O*t*Bu and C16-γE­(NHPEG_24_CO_2_Pfp)-O*t*Bu were provided by Peptech. Rink amide resin (0.3 to 0.8 mmol/g, 100–200 mesh) and Wang resin (0.4 to 0.8 mmol/g, 100–200 mesh) were purchased from Chem-Impex. Clear-OX resin was purchased from Peptide International (now Biosynth).^28^

### Peptide Synthesis

The linear peptide chains were elongated by a Liberty Microwave Peptide Synthesizer (CEM Corporation, Matthews, NC, USA). Syntheses were conducted on a 0.25 mmol scale on Fmoc-rink amide resin when the C-terminal was amide. All coupling reactions were performed with 5 equiv. amino acid in *N,N*-dimethylformamide (DMF) (0.2 M), 5 equiv of *N,N*-diisopropylcarbodiimide in DMF (0.5 M), and 5 equiv of Oxyma Pure in DMF solution (1 M). Fmoc deprotections were performed with a 20% piperidine DMF solution. Each deprotection and coupling reaction was performed with microwave energy and nitrogen bubbling. The microwave cycle was characterized by two deprotection steps: the first one was for 30 s, and the second one for 180 s. All coupling reactions were for 300 s. Doubling coupling or extended reaction time was applied to some difficult coupling steps, while time and temperature were tuned for amino acids that are easily racemized. Three DMF washing steps were performed between the coupling and deprotection steps. Fmoc-AzaTrp was reacted in NMP due to the poor solubility of AzaTrp in DMF. After coupling of the final amino acid to the peptide chain, its Fmoc group was removed with a solution of piperidine in DMF and the N-terminus was acetylated using a mixture of acetic anhydride and *N,N*-diisopropylethyl amine (DIEA) in DMF.

### Cleavage of Peptides from Rink Amide Resin

Peptides were cleaved from the resin using 5 mL of cleavage cocktail consisting of DTT/triisopropyl silane/water/trifluoroacetic acid (TFA) (2.5:2.5:2.5:92.5) per well for 3 h at room temperature. After the resin was removed by filtration, the peptides were precipitated from 25 mL of cold diethyl ether twice, dissolved in 10 mL of 50/50 acetonitrile/water solution, and freeze-dried using a lyophilizer.

### Cyclization

#### Compound **34**

Disulfide cyclization in solution was performed by adding iodine in MeOH dropwise to the crude linear peptide in acetonitrile/water until the solution remained yellow. Once the reaction was complete, excess iodine was quenched with sodium thiosulfate in water. Alternatively, Clear-OX resin^28^ was also used for disulfide formation. The product was purified by reversed-phase high-performance liquid chromatography (RP-HPLC).

#### Compound **35**

The crude linear peptide (0.1 mmol scale) was dissolved in 40 mL of a degassed 50/50 acetonitrile/water solution. The pH was adjusted to 8 with a degassed 200 mM ammonium bicarbonate aqueous solution. To this solution, 1 equiv of 1,3-dibromoxylene in 1 mL of acetonitrile was added and stirred at room temperature for 1 h to give the xylene cyclized product, purified by RP-HPLC.

#### Compound **36**

To the linear peptide on resin (0.1 mmol) in 2 mL of DMF, was added Chloroacetic acidanhydride (1 equiv) in 1 mL of DMF. The mixture was shaken at room temperature for 30 min. The resin was collected by filtration and washed with DMF and dichloromethane (DCM). The linear peptide was cleaved from the resin and precipitated from cold diethyl ether. The crude peptide was redissolved in 20 mL of 50/50 acetonitrile/water, purged with N_2_ and buffered with phosphate to pH 7. The reaction was stirred at room temperature for 1 h to give the cyclized thioether product, purified by RP-HPLC.

#### Conversion of Compound **37** to **39**

Amide cyclization was performed on-resin, utilizing orthogonal protected Lys­(Alloc) and Asp­(OAll) that were removed by Pd-catalyzed chemistry. The protocol was adapted from literature methods using Pd(0) catalyst and the mildly acidic ally scavenger *N,N*-dimethylbarbituric acid (DMBA).^29^ The peptide was treated with 0.4 equiv of Pd­(Ph_3_P)_4_ and 20 equiv of DMBA overnight in a mixture of DCM and DMF. After deprotection, amide cyclization was performed on resin with 2 equiv of hexafluorophosphate azabenzotriazole tetramethyl uranium (HATU) and 4 equiv of *N*-methylmorpholine (NMM) in NMP for 4 h at room temperature. The product was cleaved from the resin and purified by RP-HPLC.

### General Methods for PEGylation and Lipidation

The PEGylated or lipidated peptides were prepared by first synthesizing peptide **39** (Table 4) and then coupling the linkers onto the Lys^15^ residue. The conjugation procedures were described in the following section.

#### Compound **40**

To the crude peptide **39** in DMF was added 1.4 equiv of Boc-γE­(NHPEG_24_CO_2_Pfp)-O*t*Bu in DMF, followed by 2 equiv of DIEA. The resulting mixture (25 mM) was stirred at room temperature overnight. The Boc and *t*Bu protecting groups were removed by TFA/DCM (1:1) at room temperature for 2 h. DCM was removed by a rotavapor. The product was purified by RP-HPLC.

#### Compound **41**

To the peptide **39** on resin was added Fmoc-PEG_24_-NHS ester (1.5 equiv) in DMF and DIEA (2 equiv). After the conjugation reached completion, Fmoc was removed from amino-PEG_24_ by 20% piperidine in DMF. The peptide **41** was cleaved from the resin and purified by RP-HPLC.

#### Compound **42**

To the purified peptide **41** in DMF was added 1.1 equiv of palmitic acid NHS ester and DIEA (1.5 equiv). After the conjugation reached completion, peptide **42** was purified by RP-HPLC.

#### Compound **43**

To the purified peptide **39** in DMF was added 1.1 equiv of palmitic acid NHS ester and 1.5 equiv of DIEA. After the conjugation went to completion, compound **43** was purified by RP-HPLC.

#### Compound **44**

To the purified **39** in DMF was added (*t*Bu)­C18-γE­(NHPEG_24_CO_2_Pfp)-O*t*Bu (1.2 equiv), followed by addition of NMM (5 equiv), and the reaction was monitored by LC-MS. After the reaction was completed, the majority of the DMF was evaporated, and the product was precipitated with successive additions of cold diethyl ether. The peptide was dried. The *t*-butyl ester of γ-Glu and terminal C18 was then removed from the conjugate by cleavage with TFA/water/anisole (95:2.5:2.5, v/v/v), and the peptide was isolated again by precipitation in the cold ether, dried, and purified by RP-HPLC.

#### Compound **45**

Preswelled wang resin alcohol was converted to bromide by treating with PBr_3_ in DCM for 2 h at room temperature. To the resulting Wang resin bromide 2 equiv of Fmoc-Lys­(Boc)-OH in DMF was added, followed by 2.2 equiv of DIEA, and the reaction stirred overnight at room temperature. The loading of the Fmoc-Lys­(Boc)-loaded Wang resin was calculated after washing and drying. The loaded resin was taken to the linear SPPS peptide synthesis and on-resin amide cyclization (the cyclization procedure as for compounds **37** to **39**). Peptide **39** was cleaved and purified by RP-HPLC. The above 90% purity fractions were combined and taken to the next step without lyophilization. Peptide quantity was estimated by high-performance liquid chromatography (HPLC). The *t*-butyl group of C16-γE­(NHPEG_24_CO_2_Pfp)-O*t*Bu was deprotected in TFA/water (99:1) for 2 h at room temperature. The purification fractions of peptide 39 in acetonitrile/water were neutralized with Et_3_N and 1.5 equiv of the deprotected C16-γE­(NHPEG_24_CO_2_Pfp)-OH was added, and the pH was adjusted to 8 to 9 with Et_3_N. After conjugation was completed, the reaction was quenched with TFA. The reaction mixture was diluted with water and purified by RP-HPLC.

### General Methods for Peptide Characterization and Purification

#### Method A

Phenomenex Kinetex 2.6 μm C18 100 Å 2.1 × 100 mm or equivalent columns; mobile phase A = 0.1% TFA in water; mobile phase B = 0.1% TFA in acetonitrile; temperature: 25 °C; gradient: 25% B to 45% B in 12 min; flow rate: 1 mL/min; detector wavelength = 214 nM. The mass spectra (MS) were recorded on amaZon SL mass spectrometer using electrospray positive ionization mode. The cone voltage was 20 V.

#### Method B

Phenomenex Luna 2.6 μm C18 100Å 2.1 × 100 mm or equivalent columns; mobile phase A = 0.1% TFA in water; mobile phase B = 0.1% TFA in acetonitrile; temperature: 40 °C; gradient: 25% B to 65% B in 6 min; flow rate: 1 mL/min; detector wavelength = 214 nM. The mass spectra (MS) were recorded on amaZon SL mass spectrometer using electrospray positive ionization mode. The cone voltage was 20 V

#### Method C

Zorbax SB300 C3, 3.5 μm 4.6 × 150 mm or Phe-nomenex Kinetex 2.6 μm Phenyl-Hexyl Core–Shell column 4.6 × 50 mm; mobile phase A = 0.1% TFA in water; mobile phase B = 0.1% TFA in acetonitrile; temperature: 45 to 60 °C; gradient: 20% B to 60% B in 25 to 30 min; flow rate: 1 mL/min; detector wavelength = 214 or 220 nM. Electrospray ionization mass spectrometry was performed using a Waters TQP triple quadrupole instrument.

The purities of all final compounds were 95% or greater by HPLC analysis unless specified in the tabulated LC-MS data in the .

### RBC Hemolysis Assay

Inhibition of the complement classical pathway activation by a peptide was determined in a hemolysis assay using antibody-sensitized sheep erythrocytes (EA) exposed to 1% normal human serum in a GVB++ buffer. Briefly, serial dilutions of the peptide were mixed with normal human serum, EA, and GVB++ in 96-well plates at 37 °C. The samples were centrifuged to pellet the remaining intact erythrocytes, and supernatants were collected. Hemoglobin released from erythrocyte lysis was detected by measuring the optical density of the supernatant at 412 nm using a microplate reader (SpectraMax M4 from Molecular Devices. San Jose, CA, USA or Tecan SPARK, from Tecan, Männedorf, Switzerland). Percentages of hemolysis were plotted against inhibitor concentration and fitted to a standard four-parameterdose–response inhibition function (GraphPad Prism) to calculate the IC_50_.

### Surface Plasmon Resonance Assay

SPR assay was performed on a Bio-Rad ProteOn XPR36 (Bio-Rad, Hercules, CA, USA) or a Biacore 8K (formerly GE Healthcare, now part of Cytiva, Marlborough, MA, USA). Human complement C5 proteins (wild-type or R885 variants) were immobilized on a Bio-Rad GLH sensor chip docked in ProteOn or on a CM5 sensor chip docked in Biacore 8K, followed by flowing of zilucoplan or eculizumab biosimilar at varying concentrations in a 1× HEPES buffer (pH 7.4, 150 mM NaCl, 1 mM MgCl_2_, 0.005% surfactant P-20, and 1% dimethyl sulfoxide (DMSO) or a 1× phosphate-buffered saline (PBS) buffer (pH 7.4, 0.005% P-20, and 1% DMSO). The resulting SPR sensorgrams were recorded and analyzed using the software provided by the vendors to extract the association and dissociation rate constants (*k*_a_ and *k*_d_) and *K*_D_.

### Plasma Stability Assay

Monkey and human: peptide was incubated at 2 μM in human or cynomolgus monkey plasma at 37 °C in 1 mL aliquots for up to 24 h. At the end of each incubation period, a 50 μL aliquot of plasma (collected with heparin as anticoagulant) was collected and stored at −80 °C. After the final sample was collected, all samples were extracted using protein precipitation and analyzed by LC-HRMS on either a Q Exactive Orbitrap or PE SCIEX API 4000 instrument. Rat: studies were carried out in Sprague–Dawley rat plasma obtained from Bioreclamation (now BioIVT) and collected on sodium heparin. Plasma was adjusted to pH 7.4 prior to the start of the experiment. A DMSO stock solution was first prepared for the test article. An aliquot of the DMSO solution was dosed into 1 mL of plasma, which had been prewarmed to 37 °C, at a final test article concentration of 10 μM. The vials were kept in a benchtop Thermomixer for the duration of the experiment. Aliquots (100 μL) were taken at each time point and added to 96-well plates which had been prefilled with 300 μL of acetonitrile. Samples were stored at 4 °C until the end of the experiment. After the final time point was sampled, the plate was mixed and then centrifuged at 3,000 rpm for 10 min. Aliquots of the supernatant were removed, diluted 1:1 into distilled water, and analyzed by LC-MS/MS. The peak area response ratio (PARR) to the internal standard at each time point was compared to the PARR at time 0 to determine the percent of test article remaining at each time point. Half-lives were calculated by using GraphPad software, fitting to a single-phase exponential decay equation.

### PK Studies

All PK studies in preclinical species were conducted according to the highest ethical standards, in accordance with and using procedures approved by the Institutional Animal Care and Use Committee of Charles River Laboratories, Reno, Nevada IACUC on November 10, 2015 (Ethics Approval Number: 20087646). In the study, compounds were administered to male cynomolgus macaque (*n* = 2) via a single IV injection at 0.4 mg/kg. The peptide concentration was 2 mg/mL; injection volume was 0.2 mL/kg; and dosing formulation was 50 mM PBS buffer at pH 7. Blood samples were collected after the IV administration. All blood samples were collected in EDTA-coated tubes at predose, at 0.25, 1, 2, 4, and 24 h postdose, and on day 4, day 7, day 10 and day 14 postdose. All blood samples were centrifuged, and the supernatant plasma samples were shipped to Ra Pharmaceuticals and the Agilux testing facility (Worcester, MA, USA) on dry ice; samples were subsequently stored at −80 °C prior to extraction and analysis. Concentrations in cynomolgus macaque plasma were determined by LC-MS/MS assays, following a protein precipitation step. Data was acquired and processed using Analyst Version 1.5 (Applied Biosystems Sciex) and Microsoft Excel. Peak area ratios of compound to internal standard were used to determine the compound concentration in plasma. Noncompartmental PK parameters were determined from individual plasma concentration–time data using WinNonlin, version 6.3. Area under the plasma concentration–time curve (AUC) was estimated by using the linear trapezoidal linear interpolation rule.

### Crystallography

Expression of C5 TED was performed according to previously established protocols by PROTEROS. The protein was purified, comprising affinity and gel filtration chromatography steps. This procedure yielded homogeneous protein with a purity greater than 95% as judged from Coomassie stained SDS-PAGE. The purified protein was used in crystallization trails employing both a standard screen with approximately 1200 different conditions, as well as crystallization conditions identified using literature data. Conditions initially obtained have been optimized using standard strategies, systematically varying parameters critically influencing crystallization, such as the temperature, protein concentration, drop ratio, and others. These conditions are also refined by systematically varying pH or precipitation concentrations. A cryoprotocol was established using PROTEROS Standard Protocols. Crystals were flash-frozen and measured at a temperature of 100 K. The X-ray diffraction data were collected from complex crystals of C5 TED with compound **46** at the SWISS LIGHT SOURCE (SLS, Villigen, Switzerland) using cryogenic conditions. The crystals belong to the space group C2 2 2_1_. Data was processed using the programs XDS and XSCALE. Data collection and processing statistics were summarized in the . Subsequent model building and refinement was performed according to standard protocols with the software packages CCP4 and COOT. Refinement statistics were summarized in the . Coordinates and experimental data are available from the Protein Data Bank with the accession code 9Y6C.

## Supplementary Material





## Notes

Tabulated LCMS data of all compounds and LCMS spectra of representative compounds, structure of compound **46**, data collection and processing statistics for compound **46**, refinement statistics for compound **46**, PK and PD study results of compound **45** (PDF)
Molecular formula strings of all compounds (CSV)

## Abbreviations

AChR: acetylcholine receptor
aHUS: atypical hemolytic uremic syndrome
Ala: alanine
Asp: aspartic acid
AUC: area under curve
AzaTrp: 7-aza tryptophan
C5: complement component 5 protein
Chg: cyclohexylglycine
Cys: cysteine
C16: 16-carbon chain fatty acid
C18OH: 18-carbon chain diacid
DIEA: *N,N*-diisopropylethyl amine
DMBA: *N,N*-dimethylbarbituric acid
εK: epsilon lysine
Et_3_N: triethylamine
GA: geographic atrophy
γE: gamma glutamic acid
gMG: generalized myasthenia gravis
Glu: glutamic acid
GVB: gelatin veronal buffer
h: hour
HATU: hexafluorophosphate azabenzotriazole tetramethyl uronium
HEPES: 4-(2-hydroxyethyl)-1-piperazineethanesulfonic acid
*k*_a_: association rate constant
*k*_d_: dissociation rate constant
*K*_D_: binding affinity
LC-HRMS: liquid chromatography-high resolution mass spectrometry
Lys: lysine
MAC: membrane attack complex
mpk: milligram per kilogram
NHS: *N*-hydroxysuccinimide
NMM: *N*-methylmorpholine
Nvl: norvaline
PARR: peak area response ratio
PCSK9: proprotein convertase subtilisin/kexin type 9
PDB: Protein Data Bank
PEG: polyethylene glycol
Phg: phenylglycine
Pfp: pentafluorophenyl ester
PK: pharmacokinetics
PNH: paroxysmal nocturnal hemoglobinuria
RP-HPLC: reverse phase high-performance liquid chromatography
Ser: serine
SPPS: solid phase peptide synthesis
SPR: surface plasmon resonance
TED: thioester-containing domain
TIS: triisopropyl silane
Tle: *tert*-leucine.
