# Supplementary material for: Discovery of Zilucoplan: A Complement C5 Inhibitor for Treatment of Anti-Acetylcholine Receptor (AChR) Antibody-Positive Generalized Myasthenia Gravis (gMG)
Source: J Med Chem. 2025 Dec 11;68(24):25772–82. doi: 10.1021/acs.jmedchem.5c02537 (PMC12751008; doi:10.1021/acs.jmedchem.5c02537)
Supplement: Supplementary file 1 [file jm5c02537_si_001.pdf]

Supporting Information

Discovery of Zilucoplan: A Complement C5 Inhibitor for Treatment of Anti-Acetylcholine Receptor (AChR) Antibody-Positive Generalized Myasthenia Gravis (gMG)

Ping Ye,[1]* Robert P. Hammer,[2] Zhaolin Wang,[2] Ketki Dhamnaskar,[2] Michelle Hoarty,[2] Zhong Ma,[2] Guo-Qing Tang,[1] Steven J. DeMarco,[2] Alonso Ricardo[2]

[1]*UCB Bioscience, Cambridge, MA 02140, United States;* [2]*Ra Pharmaceuticals, Cambridge, MA 02140, United States*

Corresponding author's email address: ping.ye@ucb.com

Table of Contents Graphic

LC-MS data S3

Table S1. LC-MS Data for All Final Molecules S3

Representative LC-MS traces for key compounds S5

Figure S1. LC-MS trace for Compound **32**: S5

Figure S2. LC-MS trace for Compound **33**: S5

Figure S3. LC-MS trace for Compound **38** S6

Figure S4. LC-MS trace for Compound **40** S7

Figure S5. LC-MS trace for Compound **42** S7

Figure S6. LC-MS trace for Compound **43** S8

Figure S7. LC-MS trace for Compound **44** S9

Figure S8. LC-MS trace for Compound **45** S10

Compound **46** S11

Figure S9. Structure of compound **46** S11

Table S2. Data collection and processing statistics for compound **46** (registered internally as Ra30303) S11

PK and PD Study Results of Compound **45** S12

Figure S10. Compound **45** time-concentration PK profiles in monkey plasma S12

Figure S11. Compound **45** time-concentration PK profiles in monkey plasma S13

Figure S12. Compound **45** percent Hemolysis PD for Monkeys Receiving a single dose 0.4mg/kg IV (Group 1) or SC (Group 2) S13

Table S4. Compound **45** WinNonlin Calculated Pharmacokinetic Parameters following single 0.4 mg/kg IV or SC dose S14

Figure S13. Compound **45** time-concentration PK profiles in monkey plasma following seven daily 0.2 mg/kg SC doses S14

Figure S14. Compound **45** time-concentration PK profiles in monkey plasma following seven daily 4 mg/kg SC doses S15

Figure S15. Compound **45** Hemolysis PD in Cynomolgus Monkey Samples from 7 daily doses SC doses (Group 1 0.2mg/kg and Group 2 4mg/kg) S15

Figure S16. PK and PD relationship of compound **45** following 7 daily doses in monkeys S16

Figure S17. Log concentration-activity curve for compound **45** following 7 daily doses in monkeys........ S16

Table S5. For dose administration, the compound **45** was diluted to the following concentrations in the indicated dosing buffer: S17

LC-MS data

Most final tested molecules in the table were > 95% purity by various analysis methods. Any compound less than 95%, the percentage of purity is specified in the purity column (Table, S2). The methods used for analysis are detailed below:

Method A: Phenomenex Kinetex 2.6µm C18 100Å 2.1 x 100 mm or equivalent columns; mobile phase A = 0.1% TFA in water; mobile phase B = 0.1% TFA in acetonitrile; temperature: 25°C; gradient: 25% B to 45% B in 12 min; flow rate: 1 mL/min; detector wavelength = 214 nM. The mass spectra (MS) were recorded on amaZon SL mass spectrometer using electrospray positive ionization mode. The cone voltage was 20 V.

Method B: Phenomenex Luna 2.6µm C18 100Å 2.1 x 100 mm or equivalent columns; mobile phase A = 0.1% TFA in water; mobile phase B = 0.1% TFA in acetonitrile; temperature: 40°C; gradient: 25% B to 65% B in 6 min; flow rate: 1 mL/min; detector wavelength = 214 nM. The mass spectra (MS) were recorded on amaZon SL mass spectrometer using electrospray positive ionization mode. The cone voltage was 20 V.

Method C: Zorbax SB300 C3, 3.5 µm 4.6 x 150 mm or Phe-nomenex Kinetex 2.6 µm Phenyl-Hexyl Core-Shell column 4.6 x 50 mm; mobile phase A = 0.1% TFA in water; mobile phase B = 0.1% TFA in acetonitrile; temperature: 45 to 60°C; gradient: 20% B to 60% B in 25 min to 30 min; flow rate: 1 mL/min; detector wavelength = 214 or 220 nM. Electrospray ionization mass spectrometry was performed using a Waters TQP triple quadrupole instrument.

Table S1. LC-MS Data for All Final Molecules

Compound #	M/Z	MS $[M+H]^+$	MS $[M+2H]^+$	MS $[M+3H]^+$	MS $[M+4H]^+$	LC Method	RT (min)	Purity by UV
**1**	1751.77	1752.74	n/a	n/a	n/a	A	10.1	>95%
**2**	1826.76	1826.83	914.00	n/a	n/a	A	9.2	>95%
**3**	1736.79	1736.75	n/a	n/a	n/a	A	9.8	>95%
**4**	1757.81	1758.83	880.05	n/a	n/a	A	10.4	>95%
**5**	1649.81	1650.66	n/a	n/a	n/a	A	9.8	90%
**6**	1683.83	1684.76	n/a	n/a	n/a	A	10.1	>95%
**7**	1698.83	1699.56	n/a	n/a	n/a	A	10.3	>95%
**8**	1699.79	1700.49	n/a	n/a	n/a	A	9.7	92%
**9**	1649.81	1650.50	n/a	n/a	n/a	A	9.8	>95%
**10**	1625.80	1626.27	n/a	n/a	n/a	A	11.3	>95%
**11**	1683.83	1684.60	n/a	n/a	n/a	A	10.1	>95%
**12**	1649.81	1650.49	n/a	n/a	n/a	A	8.6	>95%
**13**	1715.82	1716.59	n/a	n/a	n/a	A	8.8	>95%
**14**	1673.77	1674.56	n/a	n/a	n/a	A	8.5	>95%
**15**	1747.82	1748.56	875.06	n/a	n/a	A	10.4	>95%
**16**	1767.89	1768.80	n/a	n/a	n/a	A	10.7	>95%
**17**	1767.89	1768.83	n/a	n/a	n/a	A	10.5	>95%
**18**	1767.89	1768.86	n/a	n/a	n/a	A	10.8	>95%
**19**	1767.89	1768.89	n/a	n/a	n/a	A	10.5	>95%

**20**	1767.89	1767.49	n/a	n/a	n/a	A	12.5	>95%
**21**	1563.70	1564.48	n/a	n/a	n/a	A	9.8	90%
**22**	1767.89	1768.83	n/a	n/a	n/a	A	10.6	>95%
**23**	1762.84	1762.69	n/a	n/a	n/a	A	10.9	>95%
**24**	1652.70	1653.93	n/a	n/a	n/a	A	9.7	>95%
**25**	1549.69	1550.73	n/a	n/a	n/a	A	9.8	>95%
**26**	1386.62	1387.83	n/a	n/a	n/a	A	9.5	>95%
**27**	1257.58	1258.68	n/a	n/a	n/a	A	9.6	>95%
**28**	1652.70	1653.82	827.43	n/a	n/a	A	9.8	>95%
**29**	1519.64	1520.83	n/a	n/a	n/a	A	9.3	>95%
**30**	814.36	815.96	n/a	n/a	n/a	A	8.6	>95%
**31**	798.33	799.05	n/a	n/a	n/a	A	7.9	>95%
**32**	2098.92	n/a	1050.16	n/a	n/a	A	9.7	>95%
**33**	2104.96	n/a	1053.45	n/a	n/a	A	9.8	>95%
**34**	1994.85	1994.66	998.60	n/a	n/a	A	9.3	>95%
**35**	2041.89	n/a	1021.57	n/a	n/a	A	10.0	>95%
**36**	1898.87	1898.44	950.29	n/a	n/a	A	9.8	>95%
**37**	2022.01	2021.67	n/a	n/a	n/a	A	10.2	>95%
**38**	2037.01	n/a	1019.4	n/a	n/a	C	12.3	>95%
**39**	2066.03	2065.8	1033.92	689.62	n/a	A	13.8	>95%
**40**	3322.74	n/a	1662.97	1108.92	831.78	B	4.3	>95%
**41**	3193.70	n/a	1598.46	1065.86	799.76	B	4.1	>95%
**42**	3432.93	n/a	1717.06	1145.36	859.11	B	6.1	>95%
**43**	2304.26	n/a	1153.17	769.15	n/a	C	15.5	>95%
**44**	2781.46	n/a	1392.06	928.06	n/a	B	2.6	90%
**45**	3560.97	n/a	n/a	1188.5	891.7	C	7.8	94%
**46**	2112.93	n/a	n/a	1057.35	n/a	A	10.0	>95%
n/a: not available								

Representative LC-MS traces for key compounds

Representative LC-MS traces for final compounds are shown below. Large peaks in the solvent front visible on some LCs at 214-215 nm are due to solvents and are not part of the sample. The samples were run on various instruments and the printouts for all data are not consistent, but the high level of purity of each sample is clearly demonstrated.

Figure S1. LC-MS trace for Compound **32**: >95% @ 214 nm, Method A; ESI-MS: m/z calced for [M+H]+ 2098.92; observed [M+2H]2+ 1050.16.

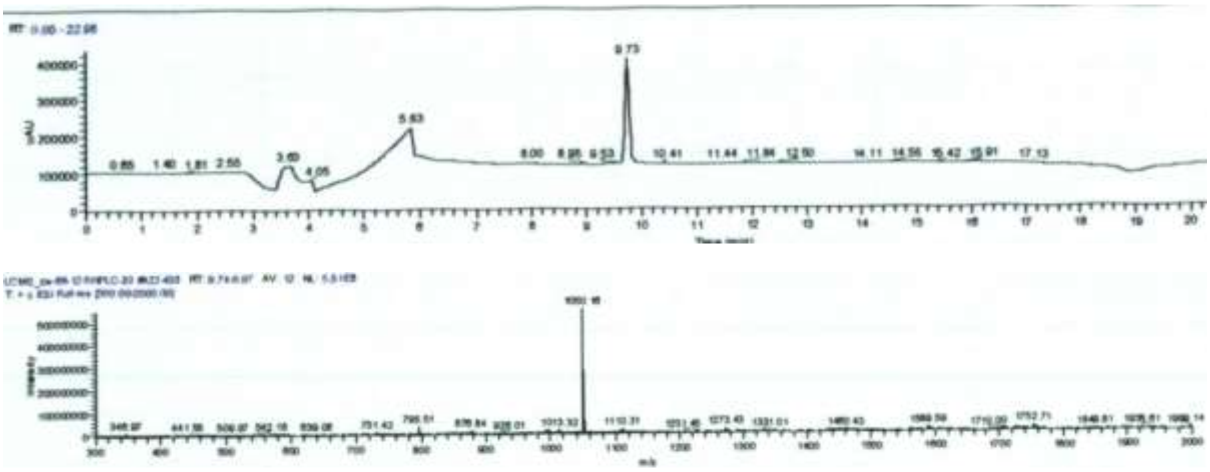

Figure S2. LC-MS trace for Compound **33**: >95% @ 214 nm, Method A; ESI-MS: m/z calced for [M+H]+ 2104.96; observed [M+2H]2+ 1053.45.

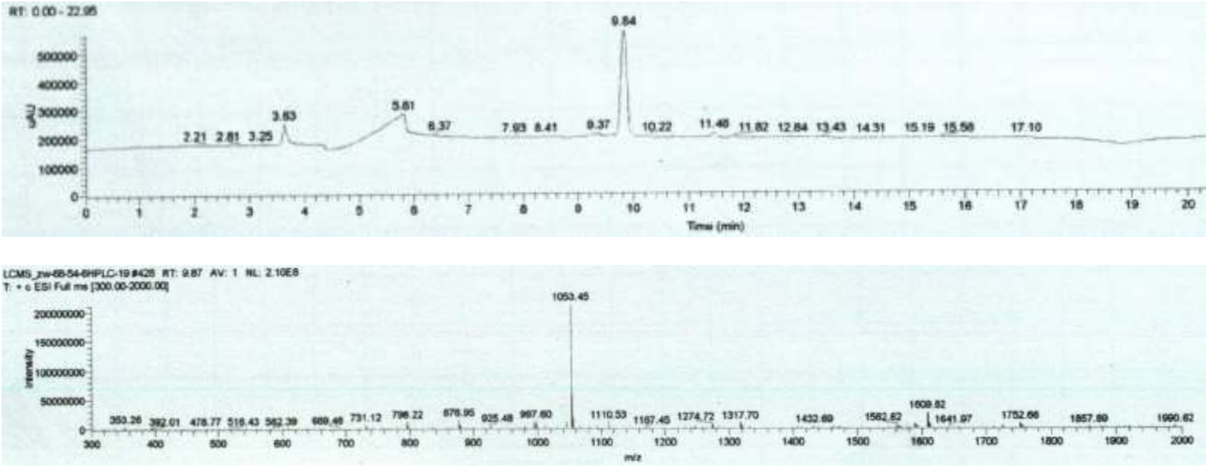

Figure S3. LC-MS trace for Compound **38**: >95% @ 215 nm, Method C; ESI-MS: m/z calced for [M+H]+ 2037.01; observed [M+2H]2+ 1019.4.

```
[illegible]
Column:           YMC C18, 4.6 X 250mm
Solvent:          A=0.1%TFA/H2O, B=0.1%TFA/ACN
Flow:             1ml/min
Detector:         215nm
Method:           C:\32Karat\Projects\Default\Method\25-55%Bin30'.met
Filename:         C:\32Karat\Projects\Default\Data\HPLC 2\AEF20-2.P02.dat
Acquisition Date:    4/3/2014 9:56:55 AM
```

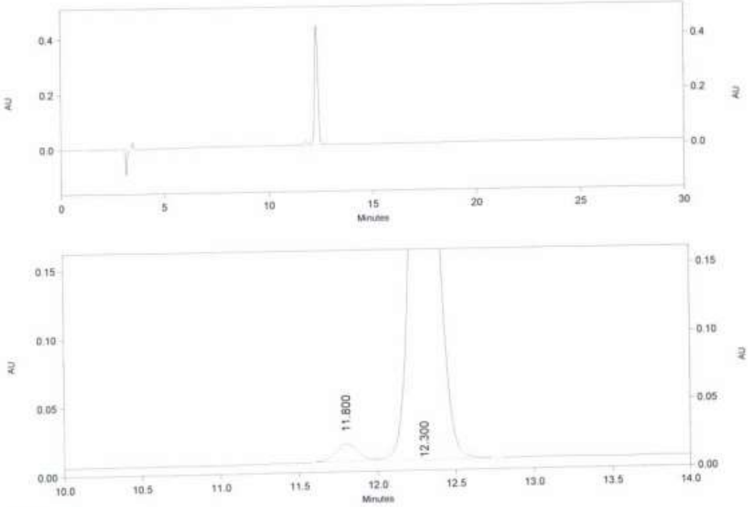

Det 166
Results

Pk #	Retention Time	Area	Area Percent	Height
1	11.80	131880	2.76	13000
2	12.30	4652685	97.24	435370
Totals		4784565	100.00	448370

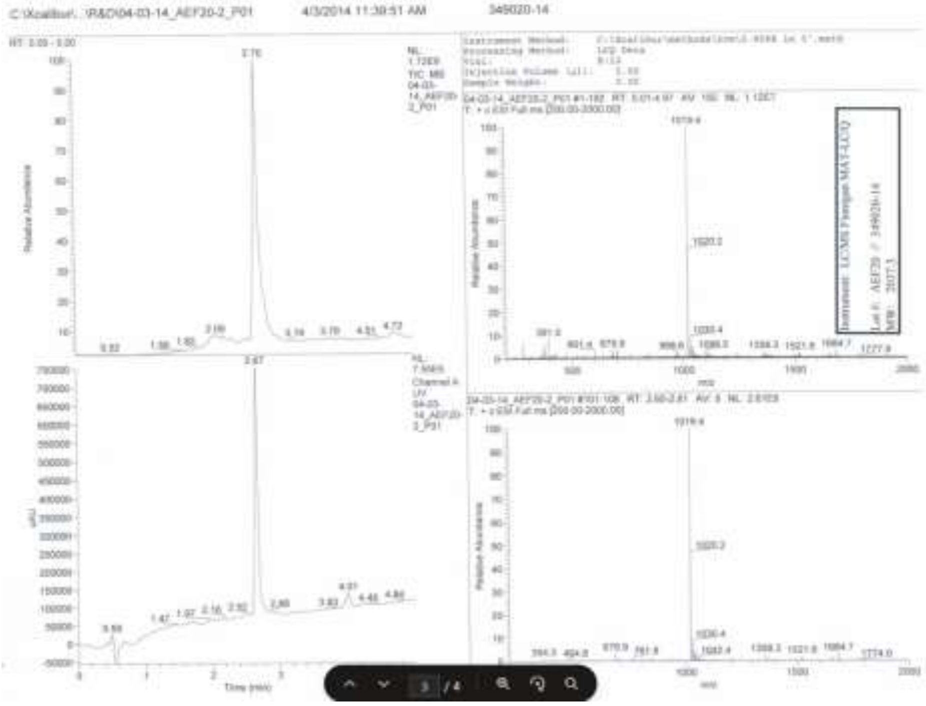

Figure S4. LC-MS trace for Compound **40**: >95% @ 215 nm, Method B; ESI-MS: m/z calced for [M+H]+ 3322.34; observed [M+2H]2+ 1162.97, [M+3H]3+ 1108.92, [M+4H]4+ 831.78.

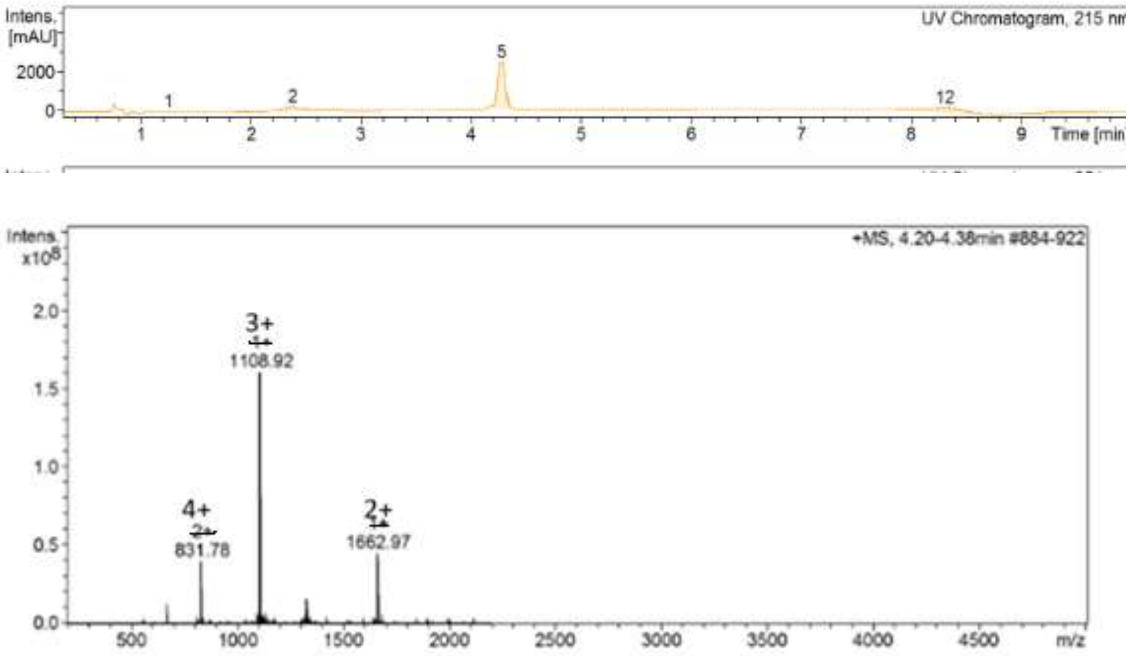

Figure S5. LC-MS trace for Compound **42**: >95% @ 215 nm, Method B; ESI-MS: m/z calced for [M+H]+ 3432.93; observed [M+2H]2+ 1717.06, [M+3H]3+ 1145.36, [M+4H]4+ 859.11, [M+5H]5+ 687.65.

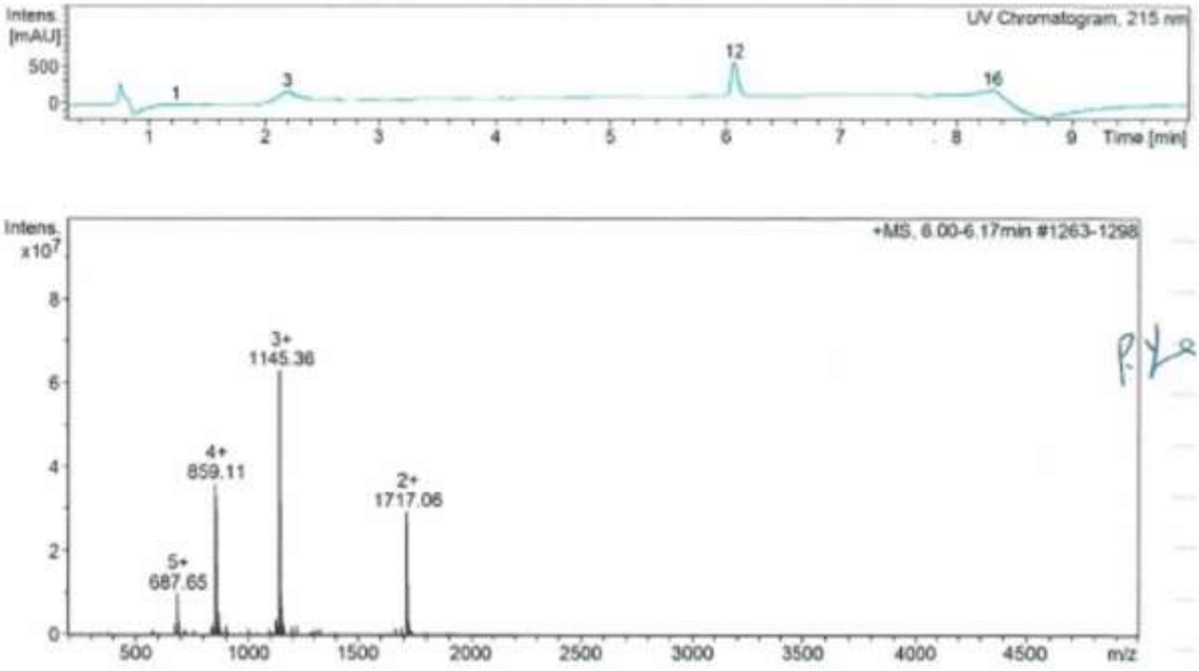

Figure S6. LC-MS trace for Compound **43**: >95% @ 215 nm, Method C; ESI-MS: m/z calced for [M+H]+ 2304.26; observed [M+2H]2+ 1153.17, [M+3H]3+ 769.15.

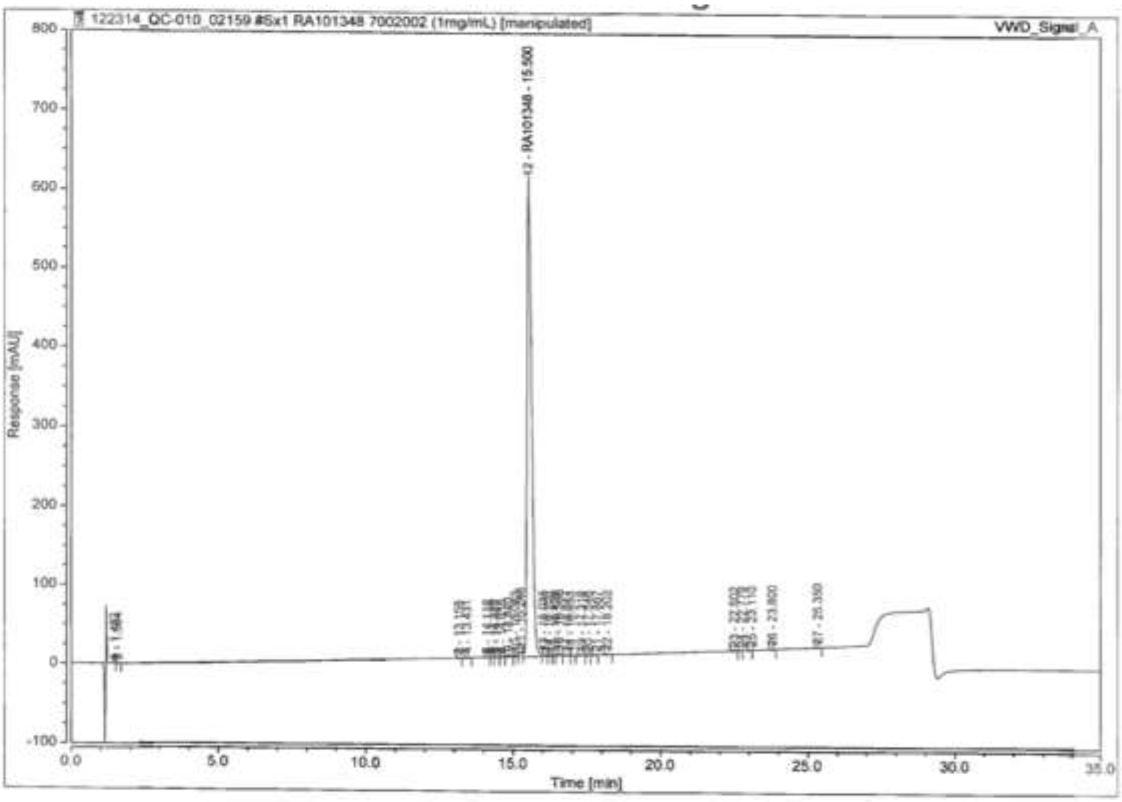

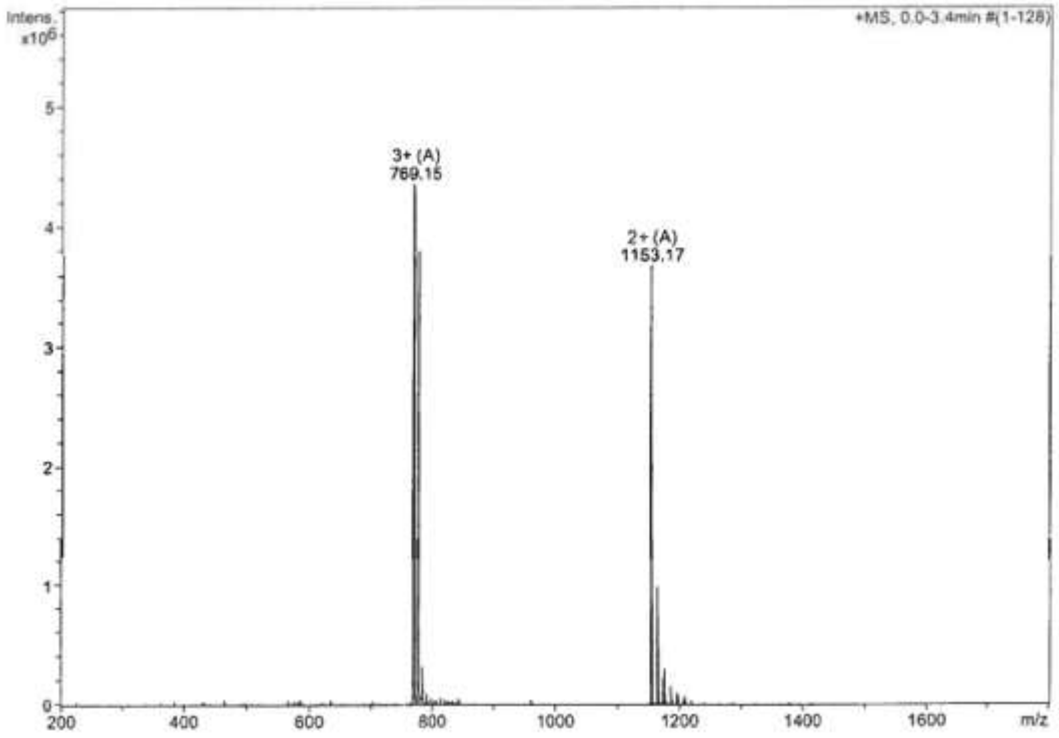

Figure S7. LC-MS trace for Compound **44**: 90% @ 215 nm, Method B; ESI-MS: m/z calced for [M+H]+ 2781.46; observed [M+2H]2+ 1392.06, [M+3H]3+ 928.06. The purity was less than 95% by HPLC due to close eluting PEG related impurities from the starting material.

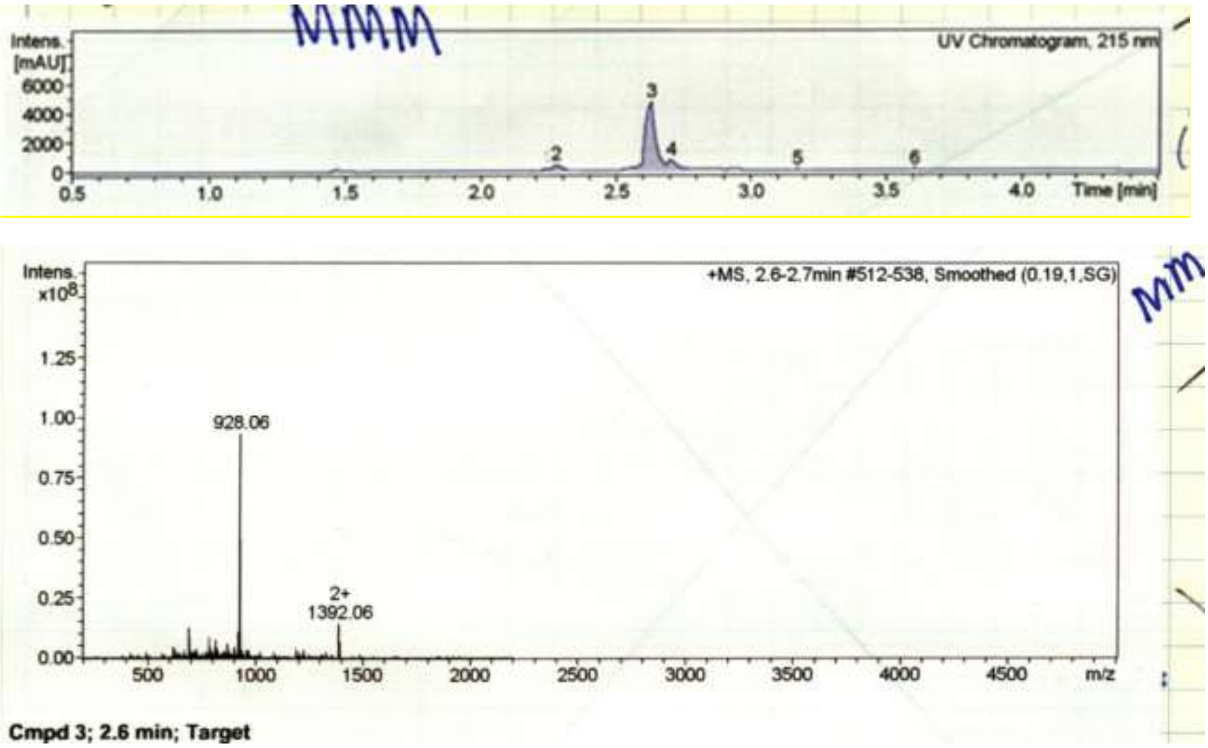

Figure S8. LC-MS trace for Compound **45**: 94.2% @ 215 nm, Method C; ESI-MS: m/z calced for [M+H]+ 3560.97; observed [M+3H]3+ 1188.5, [M+4H]4+ 891.7, [M+5H]5+ 713.6. The purity was less than 95% by HPLC due to close eluting PEG related impurities from the starting material.

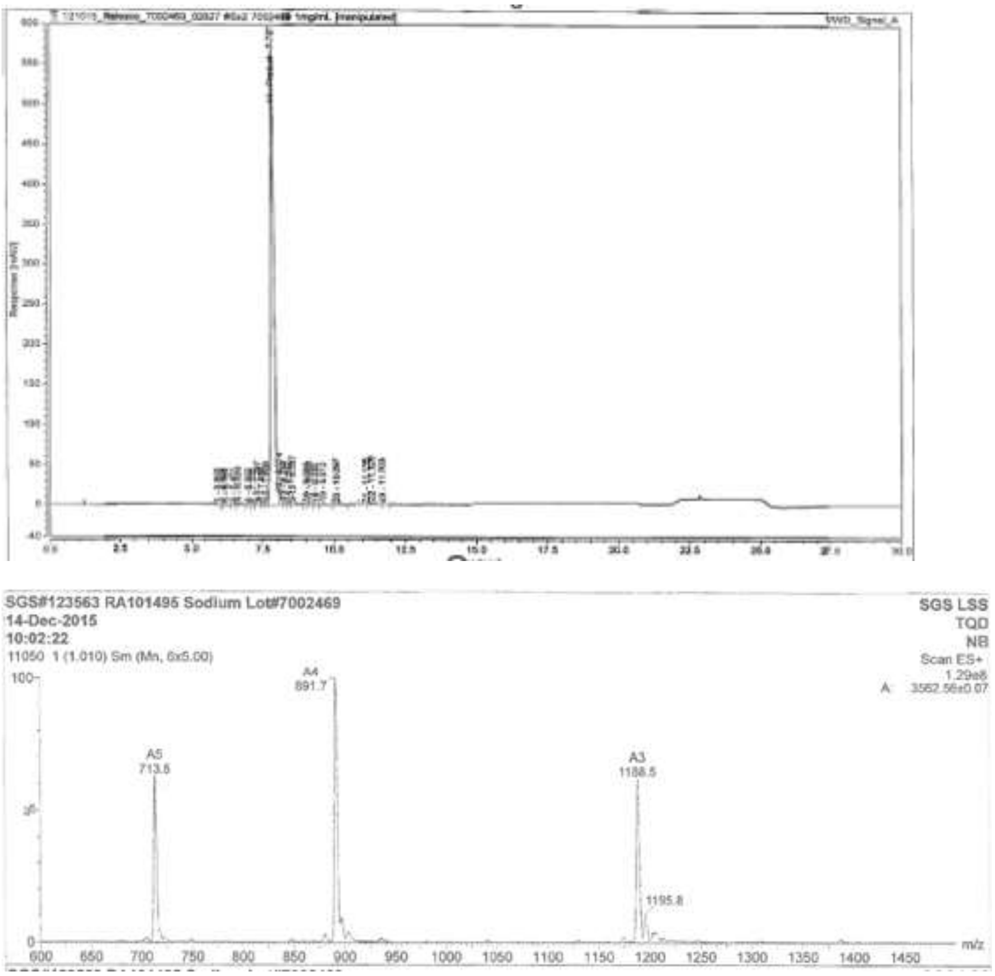

Compound **46**

Figure S9. Structure of compound **46**

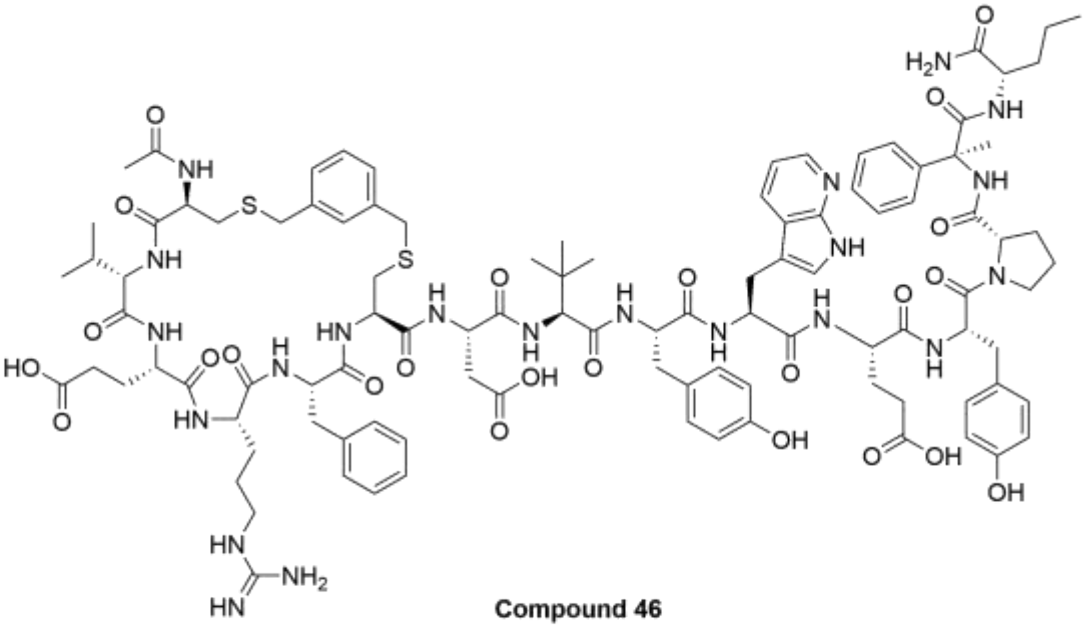

Compound 46

Table S2. Data collection and processing statistics for compound **46** (registered internally as Ra30303)

Table 2 Data collection and processing statistics for Ra30303

**Peptide**	**Ra30303**
X-ray source	PXI/X06SA (SLS[1])
Wavelength [Å]	1.00000
Detector	PILATUS 6M
Temperature [K]	100
Space group	C 2 2 2_1
Cell: a; b; c; [Å]	45.05; 112.03; 164.98
α; β; γ; [◦]	90.0; 90.0; 90.0
Resolution [Å]	2.00 (2.25-2.00)
Unique reflections	27129 (8040)
Multiplicity	4.8 (4.7)
Completeness [%]	94.3 (95.3)
R_{sym} [%][3]	14.3 (75.0)
R_{meas} [%][4]	16.0 (84.4)
Mean(I)/sd[5]	7.66 (1.89)

[1] SWISS LIGHT SOURCE (SLS, Villigen, Switzerland)

[2] values in parenthesis refer to the highest resolution bin.

[3] $$Rsym = \frac{\sum_h \sum_i^{n_h} \left| \hat{I}_h - I_{h,i} \right|}{\sum_h \sum_i^{n_h} I_{h,i}} \quad \text{with} \quad \hat{I}_h = \frac{1}{n_h} \sum_i^{n_h} I_{h,i}$$

where $I_{h,i}$ is the intensity value of the ith measurement of h

[4] $$Rmeas = \frac{\sum_h \sqrt{\frac{n_h}{n_h - 1}} \sum_i^{n_h} \left| \hat{I}_h - I_{h,i} \right|}{\sum_h \sum_i^{n_h} I_{h,i}} \quad \text{with} \quad \hat{I}_h = \frac{1}{n_h} \sum_i^{n_h} I_{h,i}$$

where $I_{h,i}$ is the intensity value of the ith measurement of h

[5] calculated from independent reflections

Table S3. Refinement statistics for compound **46**

Table 3 Refinement statistics for Ra30303[1]

**Peptide**	**Ra30303**
Resolution [Å]	82.48-2.00
Number of reflections (working /test)	25258 / 1870
R_{cryst} [%]	20.6
R_{free}[%][2]	25.3
Total number of atoms:	
Protein	2574
Water	170
Peptide	150
Deviation from ideal geometry: [3]	
Bond lengths [Å]	0.008
Bond angles [°]	1.09
Bonded B's [$Å^2$] [4]	4.5
Ramachandran plot: [5]	
Most favoured regions [%]	94.9
Additional allowed regions [%]	4.4
Generously allowed regions [%]	0.7
Disallowed regions [%]	0.0

[1] Values as defined in REFMAC5, without sigma cut-off
[2] Test-set contains 2.4 % of measured reflections
[3] Root mean square deviations from geometric target values
[4] Calculated with MOLEMAN
[5] Calculated with PROCHECK

PK and PD Study Results of Compound **45**

Figure S10. Compound **45** time-concentration PK profiles in monkey plasma

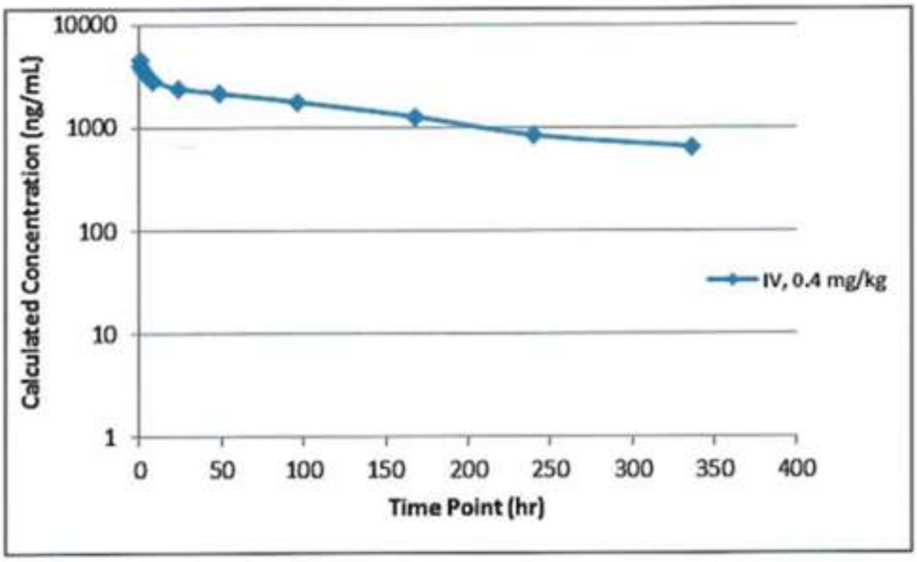

Figure S11. Compound **45** time-concentration PK profiles in monkey plasma

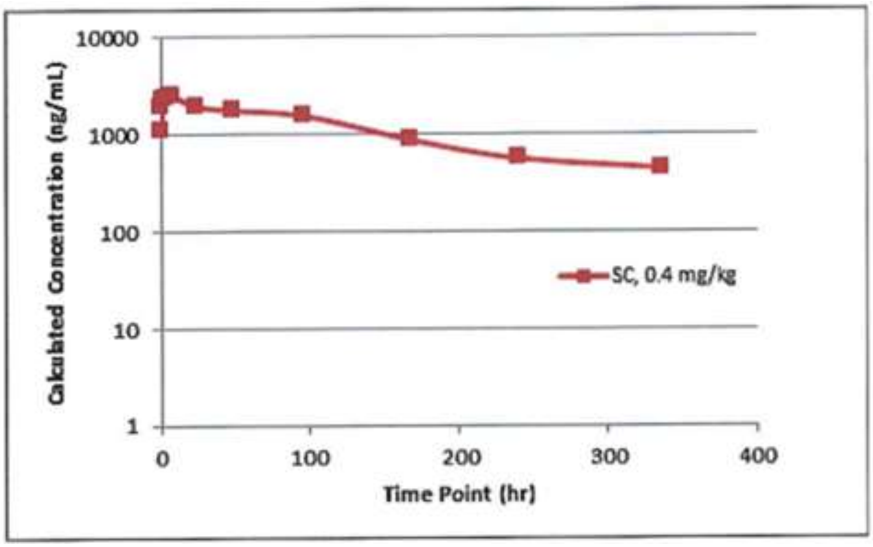

Figure S12. Compound **45** percent Hemolysis PD for Monkeys Receiving a single dose 0.4mg/kg IV (Group 1) or SC (Group 2)

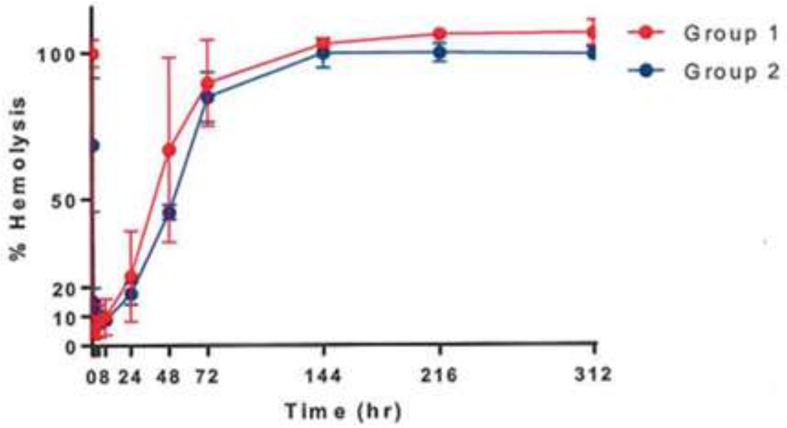

Table S4. Compound **45** WinNonlin Calculated Pharmacokinetic Parameters following single 0.4 mg/kg IV or SC dose

RoA	Dose (mg/kg)	Monkey ID	$t_{1/2}$ (hr)	T_{max} (hr)	C_{max} (ng/mL)	AUC_{0-last} (hr*ng/mL)	$AUC_{0-\infty}$ (hr*ng/mL)	%F
IV	0.4	2066-1210983M	211	0.250	4897	405975	603491	NA
		2067-1212565M	154	0.250	4594	453301	599294	NA
SC	0.4	2068-1208625M	189	8.00	2450	314873	430449	73.3
		2069-1207563M	166	8.00	2530	335762	447925	78.1

RoA	Dose (mg/kg)	Monkey ID	Vz (mL/kg)	CL (mL/min/kg)	MRT_{last} (hr)	V_{ss} (mL/kg)
IV	0.4	2066-1210983M	202	0.011	111	183
		2067-1212565M	149	0.011	114	144
SC	0.4	2068-1208625M	NA	NA	NA	NA
		2069-1207563M	NA	NA	NA	NA

Figure S13. Compound **45** time-concentration PK profiles in monkey plasma following seven daily 0.2 mg/kg SC doses

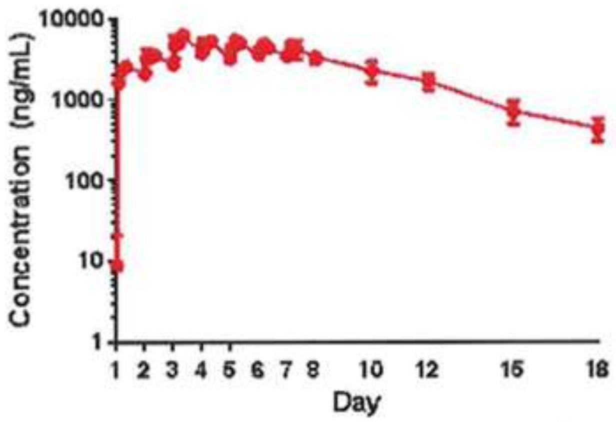

Figure S14. Compound **45** time-concentration PK profiles in monkey plasma following seven daily 4 mg/kg SC doses

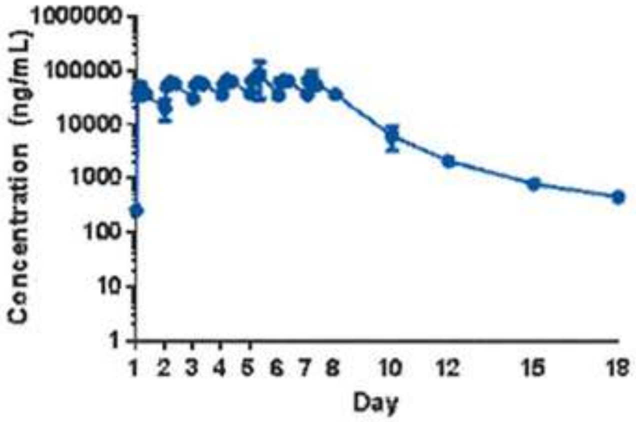

Figure S15. Compound **45** Hemolysis PD in Cynomolgus Monkey Samples from 7 daily doses SC doses (Group 1 0.2mg/kg and Group 2 4mg/kg)

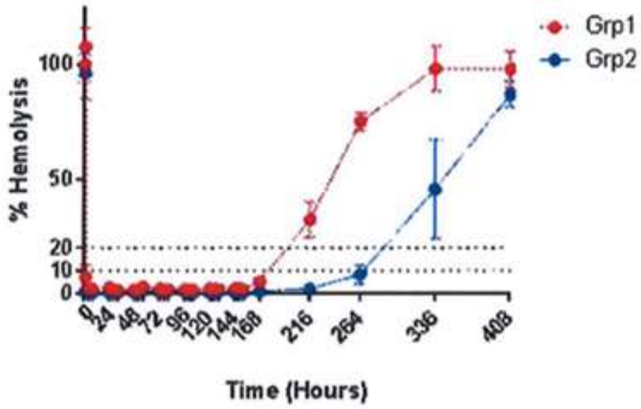

Figure S16. PK and PD relationship of compound **45** following 7 daily doses in monkeys

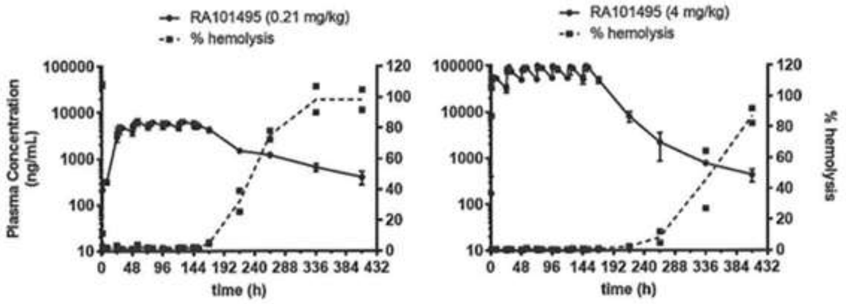

Figure S17. Log concentration-activity curve for compound **45** following 7 daily doses in monkeys

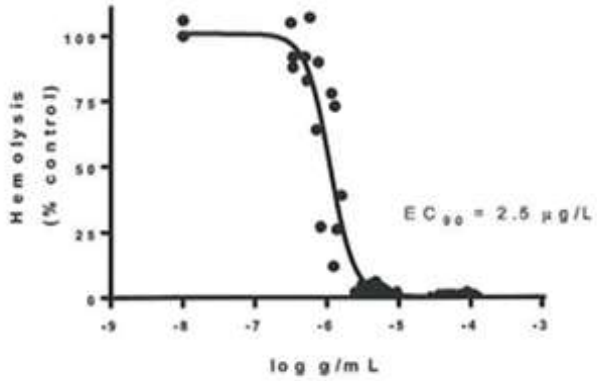

Table S5. For dose administration, the compound **45** was diluted to the following concentrations in the indicated dosing buffer:

Compound-Lot number	Route	Dose (mg/kg)	Conc. API (mg/mL)	Injection Vol. (mL/kg)	Dosing Formulation	Frequency
RA101495-4	IV	0.5	0.167	3	50 mM PBS buffer, pH 7, 5% DMSO	Single dose
RA101495-4	SC	0.5	0.167	3	50 mM PBS buffer, pH 7, 5% DMSO	Single dose
RA101495-9	SC	0.2	0.0667*	3	50 mM PBS buffer, pH 7	7 doses, daily
RA101495-9	SC	4	1.33*	3	50 mM PBS buffer, pH 7	7 doses, daily
RA101495-22	SC	0.2	1	0.2	50 mM PBS buffer, pH 7	3 doses, every 3 days
RA101495-22	SC	0.4	2	0.2	50 mM PBS buffer, pH 7	3 doses, every 3 days
RA101495-22	SC	0.6	3	0.2	50 mM PBS buffer, pH 7	3 doses, every 3 days
RA101495-22	IV	0.4	2	0.2	50 mM PBS buffer, pH 7	Single dose
RA101495-22	SC	0.4	2	0.2	50 mM PBS buffer, pH 7	Single dose

* Bulk concentration was corrected to align with revised estimated net peptide content
